# Equine histones are mobilized within equid alphaherpesvirus 1 (EHV1) replication compartments

**DOI:** 10.1128/jvi.01589-25

**Published:** 2025-11-25

**Authors:** Kristen L. Conn

**Affiliations:** 1 Department of Veterinary Microbiology, University of Saskatchewan 548643, Saskatoon, Saskatchewan, Canada; Dartmouth College Geisel School of Medicine, Hanover, New Hampshire, USA

**Keywords:** alphaherpesvirus, *Varicellovirus*, EHV1, linker histone, core histone, histone variants, FRAP, histone dynamics, viral chromatin, nucleolus

## Abstract

Equid alphaherpesvirus 1 (EHV1) is a DNA virus that causes severe disease in equids. Some strains are neurotropic and cause disease in the central nervous system, whereas others are non-neurotropic and can cause negative reproductive outcomes. Mechanisms governing EHV1 pathotype are not understood. However, EHV1 pathotypes have different infection efficiencies and replication kinetics in various cell types. They are also differentially susceptible to epigenetic inhibitors. Aside from this observation little is known about EHV1 chromatin or how its regulation influences infection. To build knowledge of EHV1 chromatin, we characterized equine histone mobility during EHV1 infection of equine cells using fluorescence recovery after photobleaching. We show that non-neurotropic or neurotropic EHV1 equally mobilized canonical (H2A, H2B, H3.1, and H4) and variant (H2A.B, H2A.Z, H2A.X, macroH2A, and H3.3) core and linker H1.2 histones. EHV1 mobilized dynamic histone populations by increasing their free pools and fast chromatin exchange. With the exception of H2A.B, all histones were most mobile within EHV1 replication compartments where EHV1 chromatin is enriched. Such mobility is consistent with highly dynamic viral chromatin. Distinct histone mobilities within EHV1- or infected-cell chromatin also indicate that different mechanisms regulate either chromatin. EHV1 mobilization of histones is similar to that reported for herpes simplex virus 1 (HSV1). This unique chromatin regulatory strategy is thus conserved among alphaherpesviruses in the *Varicellovirus* and *Simplexvirus* genera. Importantly, this conservation highlights histone mobilization as a robust chromatin regulatory strategy to promote viral genome accessibility during infection of evolutionarily distinct species.

**IMPORTANCE:**

DNA viruses are subject to chromatin regulation of their gene expression. Understanding how viruses overcome genome silencing or promote the expression of their genes is important to understand how viruses take over host cells and establish productive infection. We show that EHV1 robustly mobilizes histones within nuclear domains enriched in viral chromatin. Histone mobilization would destabilize chromatin and is consistent with the assembly of dynamic or unstable EHV1 nucleosomes. Histone mobilization is a phenomenon first described for herpes simplex virus 1 (HSV1). Thus, destabilization of chromatin by mobilizing histones is conserved across the *Varicellovirus* and *Simplexvirus* genera of alphaherpesviruses. Although this unique chromatin regulatory approach is conserved, we identified differences in histone mobilization by either virus. Knowledge of how alphaherpesviruses mobilize histones during the infection of evolutionarily distinct species will increase our understanding of viral chromatin regulation and support the development of novel therapeutics to silence viral genomes.

## INTRODUCTION

Equid alphaherpesvirus 1 (EHV1) is prevalent and highly infectious (1, 2). It establishes primary lytic replication within epithelial cells of the upper respiratory tract, disseminates throughout the body in a monocyte-associated viremia, and establishes secondary lytic replication in endothelial cells lining blood vessels (3–5). Negative reproductive outcomes, including abortion and neonatal foal death, occur when EHV1 infects uterine vasculature (6). Neurological symptoms, including equine herpes myeloencephalopathy (EHM), occur when EHV1 infects cells within central nervous system (CNS) vasculature (6). The molecular mechanisms that govern whether any EHV1 strain has neurotropism are not understood. One correlate is a single-nucleotide polymorphism in the DNA polymerase gene (open reading frame [ORF] 30) (7–9). However, this polymorphism is neither strictly associated with nor predictive of neurotropism (10–12). Additional pathotype distinctions are highlighted through their infection efficiencies and replication kinetics. Neurotropic EHV1 infection is characterized by increased replication in epithelial cells, more pronounced and extended viremia due to increased infection and faster replication within immune cells, and increased transfer to and replication within endothelial cells (3, 13–15). These differences may, in part, relate to the chromatin regulation of EHV1 gene expression. Neurotropic strains are less sensitive than non-neurotropic ones to chromatin silencing in monocytes; however, they are sensitive to chromatin silencing within epithelial cells (13, 14, 16). To characterize how EHV1 chromatin regulation relates to replication kinetics, and investigate whether it contributes to pathotype, more knowledge of EHV1 chromatin is required.

The basic chromatin unit is the nucleosome, a core histone octamer composed of two molecules of histones H2A, H2B, H3, and H4 wrapped in 147 bp of DNA (17). Linker H1 histones bind to DNA at nucleosome entry-exit sites to stabilize and compact chromatin (18, 19). Histone-histone and histone-DNA interactions within and between nucleosomes regulate chromatin compaction and stability to physically control DNA access. Decreased DNA access occurs when these interactions are stabilized, whereas increased DNA access occurs when they are de-stabilized. Multiple factors regulate nucleosome stability, including the association of chromatin-binding or -regulatory proteins, post-translational modifications (PTMs) of histones, and incorporation of histone variants. Variant histones have unique amino acid (a.a.) sequences that structurally and functionally regulate nucleosome stability and DNA accessibility (20–26). Histones H2A and H3 have distinct variants, while H2B and H4 have no somatic variants in equids.

H2A somatic variants, H2A.X, H2A.Z, macroH2A, and H2A.B, are structurally and functionally diverse (27). H2A.X most resembles canonical H2A, sharing 82% identity; however, H2A.X-containing nucleosomes are less stable (28). Furthermore, phosphorylation of H2A.X (γ-H2A.X) in response to DNA damage increases nucleosome instability to facilitate DNA access for repair (28). H2A.Z shares only 60% identity with canonical H2A, and H2A.Z-containing nucleosomes are also more unstable than H2A-containing ones (29–31). MacroH2A is a more distinct variant with only 64% similarity to H2A in its amino (N)-terminus and an additional unique linker region that connects a macrodomain to its carboxyl (C)-terminus. MacroH2A increases nucleosome stability, compaction, and inaccessibility through stronger intranucleosomal interactions (32–34). MacroH2A-containing nucleosomes therefore largely restrict DNA access (35–37). H2A.B (H2A.Bbd) is the most divergent somatic H2A variant and shares only 48% identity with canonical H2A. It has a unique N-terminal arginine-rich region, has a truncated C-terminus, and only wraps 118 bp of DNA (38, 39). H2A.B nucleosomes are very unstable and therefore enriched in highly transcriptionally active chromatin and regions of DNA synthesis (39–48).

H3 has only two somatic variants, H3.3 and centromere-specific CENPA (centromeric protein A; CenH3). H3.3 is more than 96% identical to canonical H3, and nucleosomes assembled with either histone are similarly stable. However, H3.3 synergizes with co-assembled H2A variants to further regulate nucleosome stability and DNA accessibility (31, 49). The more diverse CENPA shares only 45% sequence identity with canonical H3 and assembles in centromeric nucleosomes (50). CENPA-containing nucleosomes loosely wrap only 121 bp of DNA and form an untwisted chromatin structure to increase nucleosome accessibility and destabilize chromatin (51–53).

Histones continually undergo chromatin exchange to regulate chromatin at any given locus. Intrinsic histone exchange relates to the stability of nucleosomal interactions. Thus, H1 undergoes the fastest exchange, whereas H2A-H2B dimers peripheral in the nucleosome undergo faster exchange than H3-H4 dimers central in the nucleosome (54–64). For any given histone type, variants have inherently distinct exchange rates consistent with their effects on nucleosome stability. As such, destabilizing histones, such as H2A.Z or H2A.B, undergo faster exchange than stabilizing ones, such as macroH2A (29, 48).

External factors that alter nucleosome stability also regulate histone exchange. These factors include histone PTMs, the association of chromatin-interacting or -regulatory proteins, and processes that require DNA access (65–68). Nucleosomes fully or partially disassemble ahead of DNA or RNA polymerases to facilitate DNA access and reassemble behind them. Highly transcriptionally active chromatin, therefore, has very fast histone exchange to accommodate the necessary degree of DNA accessibility. Accordingly, highly transcriptionally active chromatin, such as nucleolar chromatin, consists of very dynamic and unstable nucleosomes. These regions often appear as “nucleosome-free” when evaluated by most common chromatin interrogation methods due to inherent nucleosome instability and DNA hyperaccessibility (69–75).

Histone exchange (mobility) can be used to inform on viral chromatin. The alphaherpesvirus herpes simplex virus 1 (HSV1) broadly mobilizes all evaluated linker and core histones with the exception of H2A.B (54–57, 76). Mobilization of histones from the cellular chromatin provides a source for those histones that assemble in HSV1 chromatin. Consistently, histone mobilization relates to HSV1 chromatin composition (56, 57). HSV1 mobilizes histones the most within viral replication compartments (RCs) where viral chromatin is enriched (76). The high mobility of histones within HSV1 RCs is consistent with their assembly in highly dynamic, unstable, and accessible nucleosomes. Measured histone mobility, therefore, relates to the remarkable hyperaccessibility of HSV1 lytic chromatin (57, 77–80).

To initiate the investigation of EHV1 chromatin, we evaluated the mobility of equine histones in EHV1-infected equine cells. To this end, we used fluorescence recovery after photobleaching (FRAP), as it is the only technique to directly measure histone mobility in living cells. Non-neurotropic or neurotropic EHV1 equally mobilized all evaluated canonical (H2A, H2B, H3.1, and H4) and variant (H2A.B, H2A.Z, H2A.X, macroH2A, and H3.3) core histones and linker histone H1.2. We show that, with the exception of H2A.B, all histones were most mobile within RCs where EHV1 chromatin is enriched. Mobilization of histones within RCs increased their steady-state free pool levels and increased their fast chromatin exchange. Histones within clearly identifiable RCs were more mobile than within mock-infected nucleoli. This indicates that EHV1 chromatin is even more dynamic than nucleolar chromatin. Viral regulation of histone mobility is a unique chromatin regulatory mechanism to destabilize viral chromatin. Importantly, this mechanism is conserved across the *Varicellovirus* and *Simplexvirus* genera of alphaherpesviruses and among viruses with evolutionarily distinct host species.

## MATERIALS AND METHODS

### Cells and virus

EDerm cells (NBL-6; ATCC) were maintained in Dulbecco’s modified minimum Eagle’s medium (DMEM, Gibco 11885-084) supplemented with 10% fetal bovine serum (FBS, Corning 35-077-CV) at 37°C in 5% CO_2_. EHV1 strains, R08-8428 and D08-8315, were generous gifts from Dr. Vikram Misra (University of Saskatchewan). Strain D08-8315 has ORF 30 G2254 (D752) associated with the neurological EHV1 pathotype (8, 9). This strain was isolated from a 17-year-old female quarter horse with neurological deficits (Prairie Diagnostic Services [PDS], Saskatoon). Strain R08-8428 has ORF 30 A2254 (N752) associated with non-neurotropic EHV1 pathotype (8, 9). No information regarding this strain isolation is available (PDS, Saskatoon).

### Viral stock preparation and titration

Viral stocks were prepared and titrated in EDerm cells. Briefly, cells at approximately 40% confluence were infected with 0.01–0.05 plaque-forming units (PFU) per cell diluted in a minimal volume of 4°C DMEM. Following inoculum addition, the cells were incubated at 37°C in 5% CO_2_ for 1 h with rocking and rotating every 5–10 min. The inoculum was then removed, and the cells were washed twice with 4°C phosphate-buffered saline (PBS; 150 mM NaCl, 1 mM KH_2_PO_4_, 3 mM Na_2_HPO_4_, pH 7.4) prior to the addition of fresh 37°C DMEM supplemented with 10% FBS. Cells incubated at 33°C in 5% CO_2_ until greater than 95% cytopathic effect was observed. Infected cells were then harvested and pelleted by centrifugation at 3,214 × *g* for 30 min at 4°C. Extracellular virions were isolated from the resulting supernatant by centrifugation at 10,000 × *g* for 2 h at 4°C. Intracellular virions were released from the cell pellet by the disruption of cell membranes through three rapid freeze-thaw cycles in a dry ice-EtOH bath and 37°C water bath, respectively. Cellular debris was pelleted by centrifugation at 5,500 × *g* for 30 min at 4°C. Intra- and extra-cellular virions were combined for EHV1 stocks.

For EHV1 stock titration, EDerm cells were seeded in 12-well tissue culture plates such that they would be 50%–60% confluent for infection. The EHV1 stock was serially diluted 10-fold in 4°C DMEM, and cell monolayers were overlayed with a minimum volume of serial dilutions. Cells incubated at 37°C in 5% CO_2_ for 1 h with rocking and rotating every 5–10 min. Dilutions were removed; the cells washed twice with 4°C PBS and overlayed with 37°C 0.5% (wt/vol) methylcellulose in DMEM supplemented with 5% FBS. Infected cells incubated at 37°C in 5% CO_2_ until well-defined plaques were visible in the monolayer. Cells were fixed and stained by the addition of 1% (wt/vol) crystal violet in 17% (vol/vol) MeOH and incubated at room temperature for a minimum of 24 h. Titrations were washed by gentle agitation in ambient water and air-dried at room temperature prior to counting plaques.

### Plasmids

Constructs encoding green fluorescent protein (GFP) fused to the N-terminus of histones H2A.X, H2B, H3.3, H3.1, and H4, and GFP fused to the C-terminus of H2A.Z were generous gifts from Dr. Luis Schang (Cornell University) (55–57, 76). The amino acid sequences of equine and human histones H2A.Z.1 (XP_023493427, NP_002097), H2B (XP_001497452, NP_003514), H3.3 (NP_001356113, NP_001365974), H3.1 (NP_001356115, NP_001368928), and H4 (XP_001496752, NP_001029249) are identical. The DNA sequence encoding H2A.Z.1 (H2A.Zv) was PCR amplified from pH2A.Z.1-GFP using primers H2A.Z F and H2A.Z R (Table 1) with flanking BglII and HindIII restriction sites, respectively. Amplified DNA was ligated in-frame with GFP in pEGFP-C1 (a generous gift from Dr. Luis Schang, Cornell University) to create pEGFP-H2A.Z. Equine (XP_023500737) and human (NP_002096) H2A.X differ at amino acid 131 (Fig. S1). Human H2A.X 131S was changed to equine 131A by site-directed mutagenesis of pEGFP-H2A.X using primers H2A.X S131A F and H2A.X S131A R (Table 1).

**TABLE 1:** PCR primers^*a*^

Primer	Sequence (5′ to 3′)
H2A.Z F	AAAA**AGATCT**ATGGCTGGCGGTAAGGCTGGA
H2A.Z R	AAAA**AAGCTT**TTAGGTGGCGACCGGTGGATC
H2A.X S131A F	TGGGGCCGAAGGCGCCCGCGGGCGGCAAGA
H2A.X S131A R	CCTTCTTGCCGCCCGCGGGCGCCTTCGG
H2A F	TTTT**AGATCT**ACCATGTCTGGACGTGGCAAGCA
H2A R	TTTT**GTCGAC**AGCCCTTACTTGCCCTTAGCTT
H1 F	TTTT**AGATCT**GAACCTTTGGTGTTCAGCATG
H1 R	TTTT**AAGCTT**AAGCAGACGTTCGACCTACTT

^*a*^ Restriction enzyme recognition sequences are bolded.

Equine and human H2A are identical (XP_001505083, NP_003501). The sequence encoding H2A was PCR-amplified from U2OS (human osteosarcoma; ATCC HTB-96) genomic DNA using the primers H2A F and H2A R (Table 1). Amplified DNA was ligated in-frame with GFP in pEGFP-C1 using BglII and SalI restriction sites to create pEGFP-H2A. DNA sequences encoding equine macroH2A (XP_023473434.1) and equine H2A.B (XP_023489462.1) were ordered as gBlocks from Integrated DNA Technologies (IDT; Table 2; Fig. 11A; Fig. S1). Flanking BglII and KpnI (macroH2A) or BglII and HindIII (H2A.B) restriction sites were used for in-frame ligation with GFP in pEGFP-C1 to create pEGFP-macroH2A or pEGFP-H2A.B, respectively. The DNA sequence encoding equine linker histone H1.2 (XM_005603676) was PCR-amplified from EDerm genomic DNA using the primers H1.2 F and H1.2 R (Table 1; Fig. S1). Amplified DNA was ligated in-frame with GFP in pEGFP-C1 using BglII and HindIII restriction sites to create pEGFP-H1.2.

**TABLE 2:** Synthetic DNA sequences^*a*^

gBlock	Sequence (5′ to 3′)
MacroH2A	TTTTTT**AGATCT**ATGAGTAGCCGTGGTGGAAAGAAAAAGAGTACGAAAACCTCACGCTCTGCCAAGGCCGGAGTCATCTTCCCCGTCGGTCGCATGCTGCGTTATATCAAAAAGGGTCACCCTAAGTACAGAATTGGGGTCGGTGCTCCCGTTTATATGGCTGCCGTGCTGGAATATCTGACAGCGGAGATTCTGGAGCTGGCCGGGAACGCCGCACGTGATAACAAGAAAGGAAGGGTTACCCCCAGGCACATCCTCCTGGCCGTCGCCAACGACGAGGAACTGAATCAGCTCCTGAAGGGCGTGACGATCGCCAGCGGGGGTGTGCTTCCTAACATTCACCCTGAGTTGCTGGCTAAGAAACGTGGCAGCAAAGGCAAGTTGGAGGCCATTATCATGCCTCCCCCGGCTAAGAAAGCTAAGTCTCCGTCTCAGAAAAAGCCCGTGTCTAAGAAAGCCGGAGGCAAAAAGGGAGCCCGCAAGTCCAAAAAGCAGGGCGAGGTTTCTAAGGCAGCTTCCGCTGACTCTACTACCGAGGGCACCCCCGCCGATGGCTTCACCGTACTCAGCACCAAATCTTTGTTTCTTGGCCAGAAGTTGAATCTGATCCACAGCGAGATCAGTAACCTGGCTGGCTTCGAAGTGGAGGCGATCATTAATCCCACCAACGCCGACATCGACCTGAAAGATGACTTGGGCAACACTCTGGAGAAAAAGGGTGGGAAGGAGTTCGTGGAGGCTGTGCTCGAGTTGCGCAAAAAGAACGGCCCTCTCGAGGTCGCTGGCGCTGCCCTGTCTGCCGGCCACGGCTTGCCTGCTAAGTTTGTAATCCACTGCAACTCTCCAGTGTGGGGCGTGGACAAGTGCGAGGAACTGTTGGAGAAAACTGTCAAGAACTGCCTCGCACTGGCCGATGACAAAAAGCTCAAGAGTATCGCCTTTCCCAGTATCGGTAGCGGCCGCAACGGGTTTCCAAAACAGACAGCCGCGCAGCTGATCCTGAAAGCCATCTCTAGCTACTTCGTGTCCACTATGTCATCCTCTATCAAGACCGTTTATTTCGTACTGTTCGATTCTGAATCTATCGGTATTTATGTGCAGGAGATGGCGAAGCTTGATGCAAACTAG**GGTACC**TTTTTT
H2A.B	TTTTTT**AGATCT**ATGCCAGGCAATCGGTCCCGCCGTCGCCGTGGTAGCTCCCATGGCCAGGGTCGGCCTCGCTCACGTACAGCGCGCGCTCAACTTCTGTTCTCCGTCTCTTGGGTGGAGCACTTGCTCAGGGAAGGCCACTATGCACGCAGGTTGTCTCCGTCTGCACCTGTCTTTCTGGCCGCTGTCATTCAATACCTGACCGCCAAGGTGCTGGAACTGGCAGGCAATGAAGCATGCAAGTCCGGCCGCCGGAGGATCACTCCAGAATTGGTGGATATGGCTGTGCACAATAACGCTCTCCTGTCTGGGTTCTTTGGCGCGACAACCATCAGCCAGGTGGCCCCTGGTAGAGAGTAG**AAGCTT**TTTTTT

^*a*^ Restriction enzyme recognition sequences are bolded.

All plasmid sequences were verified by Sanger sequencing (Sanger Sequencing Platform, CRCHU de Québec-Université Laval, CHUL). Amino acid sequence comparison of equine and human histones H2A.B, H2A.X, macroH2A, and H1.2 are depicted in Fig. 11A (H2A.B) and Fig. S1 (H2A.X, macroH2A, H1.2).

### Transfection

EDerm cells (2.0–2.6 × 10^5^) were seeded in 6-well tissue culture plates at least 12 h prior to transfection. Plasmid DNA was transfected using Lipofectamine 3000 Transfection Reagent (Invitrogen, L3000001). Briefly, for each well to be transfected, 4–6 µg of plasmid DNA was combined with 4–6 µL of P3000 reagent in 100 µL of 4°C DMEM in a microfuge tube. In a separate microfuge tube, 1 µL of Lipofectamine 3000 was added to 100 µL of 4°C DMEM. Following a 5 min incubation at room temperature, the plasmid DNA-P3000 mix was added to the Lipofectamine mix. The combined transfection mix incubated at room temperature for an additional 30 min before the volume was brought to 800 µL with room temperature DMEM. Cell medium was removed, and the cells were overlayed with the transfection mix. Cells incubated at 37°C in 5% CO_2_ for 5–6 h, and then the transfection mix was removed and replaced with fresh 37°C DMEM supplemented with 10% FBS. Transfected cells were incubated in 5% CO_2_ at 37°C for at least 36 h, typically more than 48 h, prior to seeding for infection and FRAP analysis. EDerm cells have low transfection efficiency (<10%). Exogenously expressed GFP- equine histones are expressed to lower levels than endogenous ones, consistent with other studies showing transiently expressed exogenous histones with N-terminal GFP fusions to be nuclear, assemble in chromatin, and have lower expression than endogenous histones (Fig. S2) (44, 48, 54–57, 76).

### EHV1 infection

Transfected EDerm cells were seeded (4 × 10^5^) on 18 × 18 mm coverslips in 6-well tissue culture plates for FRAP analysis. Seeded cells were incubated at 37°C in 5% CO_2_ for at least 7 h prior to infection. The inoculum was prepared by diluting purified EHV1 stocks in 4°C DMEM. For mock infections, 4°C DMEM was used. The cells were overlaid with 400 µL of inoculum containing 10 PFU/cell of EHV1 strain R08-8428 (non-neurotropic) or strain D08-8315 (neurotropic). This multiplicity was used to ensure that all cells were infected, and it is lower than that used in similar studies to evaluate HSV1 mobilization of histones (30 PFU/cell) (54–56, 76). Following inoculum addition, the cells were incubated at 37°C in 5% CO_2_ for 1 h with rocking and rotating every 5–10 min. The inoculum was removed; the cells washed twice with 4°C PBS and overlayed with fresh 37°C DMEM supplemented with 10% FBS. Infected cells were incubated at 37°C in 5% CO_2_ until they were subjected to FRAP analysis.

### FRAP

Histone mobility was evaluated between 5 and 6 hpi as described previously (54–56, 76). Briefly, a coverslip was mounted on a slide and put on a 37°C stage on a Zeiss LSM 700 inverted confocal microscope (54). Cells were viewed using a Plan-Apochromat 40×/ 1.4 oil DIC objective lens. FRAP was performed using an Argon laser (488 nm) with maximum pinhole size. Whole cell imaging was performed at 2%–3% laser intensity, whereas photobleaching was achieved with 30–35 iterations at 95%–100% laser intensity. Two circular regions of equal volume, one within a nucleolus or EHV1 replication compartment of a mock- or EHV1-infected cell, respectively, and one within the surrounding cellular chromatin of the same cell were photobleached (as depicted in Fig. 3A). Thirty to forty-five differential interference contrast (DIC) and fluorescent images were collected at 0.9 s intervals from before and after photobleaching. The fluorescence of the photobleached regions at each time was normalized to the total nuclear fluorescence at that same time. The normalized fluorescence of each photobleached region at each time is presented as a ratio to its normalized fluorescence before photobleaching. Measured fluorescence recovery is therefore independent of GFP-histone expression levels within each individual cell nucleus. Fluorescence of the photobleached regions recovers as bleached GFP-histones within them exchange with fluorescent GFP-histones outside them. FRAP was measured for 30–45 s, as we were interested in measuring the mobility of the most dynamic histone populations. Any potential contribution of newly synthesized and nuclear imported GFP-histones to fluorescence recovery is also negated by this short analysis time. A single slide was used for less than 1 h. Typically, 8–10 cells per condition (mock-, R08-8428- or D08-8315-infected) from at least four independent experiments were evaluated. Cells that had obvious nuclear distortion or aberrant GFP-histone expression were excluded from the analysis.

The normalized fluorescence intensity of photobleached nuclear regions at the first time after photobleaching was used as a surrogate measure for the level of histones in the free pool (not bound in chromatin and diffusing in the nucleoplasm). The slope between normalized fluorescence at the first and second times after photobleaching, representing the initial rate of fluorescence recovery, was used as a surrogate measure for fast chromatin exchange (histones weakly assembled in chromatin). The slope of normalized fluorescence recovery from 15 to 30 s after photobleaching, representing the slow rate of fluorescence recovery, was used as a surrogate measure for slow chromatin exchange (histones stably assembled in chromatin). For linker histone H1.2, the time to recover 50% normalized fluorescence in the photobleached regions (*T_50_*) was used as a summary measure of weak chromatin interactions. Mobilization and *T_50_* inversely relate.

For each independent experiment, the level of free histone, fast or slow chromatin exchange, or *T_50_* (for H1.2) of each photobleached region within each individual mock-, R08-8428-, or D08-8315-infected cell were normalized to their average values for mock-infected cell chromatin from the same experiment. This accounts for any potential differences in photobleaching efficiency on any given day.

### 5-ethynyl 2′-deoxyuridine (EdU) labeling and confocal microscopy

EDerm cells seeded (9 × 10^4^) on glass coverslips in 24-well tissue culture plates incubated at 37°C in 5% CO_2_ at least 7 h prior to infection. Cells were mock-infected or infected with 10 PFU/cell of non-neurotropic or neurotropic EHV1 as described in “EHV1 Infection” except 150 µL inoculum was used. Nascent DNA was labeled and detected using a Click-iT Plus EdU Cell Proliferation Kit for Imaging, AlexaFluor 647 dye kit as per the manufacturer’s instructions (ThermoFisher, C10640). Briefly, the cells were incubated in 15 µM EdU from 4.5 to 5.5 hpi. The cells were then washed with 4°C PBS, fixed with 4% formaldehyde in PBS for 15 min at room temperature, and washed three times with room temperature 3% bovine serum albumin (BSA) in PBS prior to EdU detection. Following the EdU detection reaction, the cells were washed twice with 3% BSA in PBS and counterstained with Hoechst 33342 (diluted 1:2,000 in PBS) at room temperature for 15 min. The cells were washed twice in PBS and mounted on slides for imaging on a Zeiss LSM 700 inverted confocal microscope.

### Western blot

Primary rabbit antibodies used were anti-GFP (Abcam, ab290), anti-histone H2B (Abcam, ab1790), anti-histone H3 (Abcam, ab1791), and anti-histone H4 (Abcam, ab177840). The secondary antibody used was goat anti-rabbit IRDye 680 (Abcam, ab216777).

EDerm cells were transfected as described in the section “Transfection.” To replicate transfected cell treatment prior to FRAP analysis, at least 30 h after transfection cells were seeded (2 × 10^5^) in 12-well tissue culture plates and incubated at 37°C in 5% CO_2_ overnight. Transfected cells were then washed with 4°C PBS and lysed and collected in SDS-PAGE loading buffer with 4 M urea and 50 mM dithiothreitol. Whole-cell lysates were immediately snap-frozen on dry ice and stored at −20°C until use. Proteins were resolved using standard 10% Tris-tricine SDS-PAGE and electrotransferred onto 0.2 µM nitrocellulose membranes (Amersham, 10600001) for 160 min at 250 mA in Towbin buffer (25 mM Tris, 192 mM glycine, 20% [vol/vol] MeOH). Membranes were blocked in 0.2% iBlock (Thermofisher, T2015) in PBS for 1 h at room temperature on a roller apparatus.

Membranes were incubated with anti-GFP antibodies diluted 1:1,000 in 0.2% iBlock in PBST overnight at 4°C on a roller apparatus. Subsequent steps were performed on a roller apparatus at room temperature. Membranes were washed three times in PBST for 10 min each and then incubated in secondary antibody diluted 1:20,000 in 0.2% iBlock in PBST for 1 h. Membranes were then washed three times in PBST for 10 min each, once in PBS for 5 min, and rinsed in Milli-Q H_2_O prior to imaging on a LiCor Odyssey infrared imager.

Membranes were then cut and incubated with individual anti-histone primary antibodies diluted 1:1,000 in 0.2% iBlock in PBST for 2 h. Following three 10 min washes in PBST, the membranes were incubated in secondary antibody diluted 1:20,000 in 0.2% iBlock in PBST for 1 h. Membranes were washed three times in PBST for 10 min each, once in PBS for 5 min, rinsed in Milli-Q H_2_O, and imaged.

### Image preparation

Fluorescent images were analyzed using Zeiss Zen black. DIC and fluorescent image brightness and contrast were altered for figure preparation with FIJI (81).

### Statistical analysis

Statistical significance was tested using single-factor ANOVA. For comparisons where ANOVA identified differences, samples were evaluated pairwise post hoc using Tukey Kramer analysis to identify those samples that differed. Student’s two-tailed *t*-test was used for pairwise comparison of canonical and variant histone mobilities.

## RESULTS

### EHV1 mobilizes equine core histones H2B and H4

**Fig 1:**
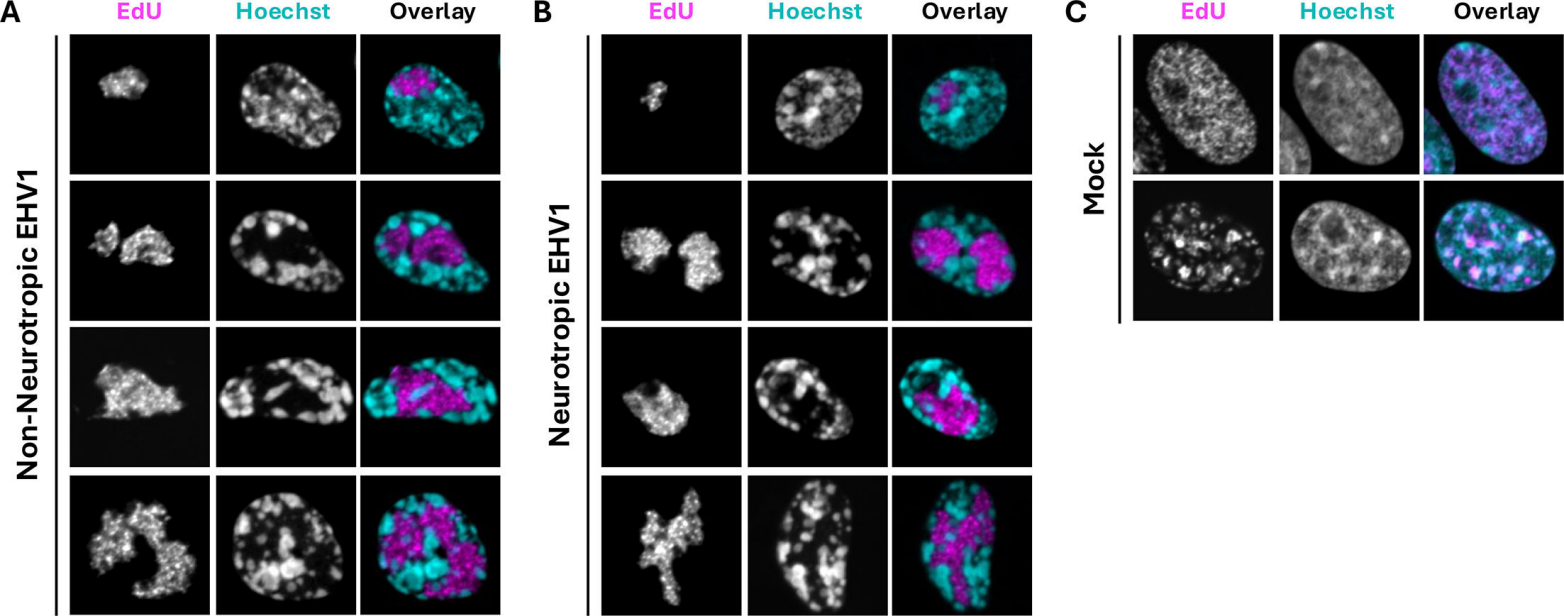
EHV1 RCs in equine cells have typical alphaherpesvirus morphology. Digital fluorescent micrographs show the nucleus of EDerm cells labeled with 5-ethynyl 2′-deoxyuridine (EdU). Cells were infected with 10 PFU per cell of non-neurotropic (**A**) or neurotropic (**B**) EHV1 or mock-infected (**C**). Nascent DNA was labeled with EdU from 4.5 to 5.5 hours post-infection (hpi), and then cells were fixed. Nuclei were counterstained with Hoechst. Images of EdU (left panels) and Hoechst (middle panels) presented in grayscale and overlayed image (right panels) in magenta (EdU) and cyan (Hoechst) for visibility.

We first evaluated mobility of equine core histones H2B and H4, as they have no somatic variants in equids that could be differentially mobilized. Furthermore, H2B represents the more mobile H2A-H2B heterodimers flanking nucleosomes, and H4 represents the more stable H3-H4 heterodimers central to nucleosomes (17, 63). We intended to comparatively evaluate histone mobility within nuclear regions enriched for EHV1 or cellular chromatin as in similar studies of HSV1 histone mobilization (76). However, as FRAP measures histone mobility in living cells, it was not possible to use immunostaining to identify EHV1 RCs, and the expression of fluorescent viral fusion protein(s) could interfere with FRAP analyses. We therefore first used 5-ethynyl 2´-deoxyuridine (EdU) to label nascent DNA and evaluate EHV1 RC morphology in fixed equine cells (Fig. 1). As expected, non-neurotropic or neurotropic EHV1 RCs had amorphous morphology characteristic of alphaherpesvirus RCs (Fig. 1A and B). Infection with either strain produced RCs of various shapes and sizes, with some occupying most of the nucleus (Fig. 1A and B). Also, as expected, EHV1 RCs occupied nuclear regions largely depleted of cellular chromatin, shown by Hoechst labeling of cellular DNA. Interestingly, cellular chromatin was highly condensed in EHV1-infected cells (Fig. 1; compare Hoechst-labeling in EHV1- vs. mock-infected cells). EdU labeling in mock-infected cells was visually distinct from that within EHV1-infected ones, and EdU was largely not excluded from Hoechst-enriched regions within them (Fig. 1C).

**Fig 2:**
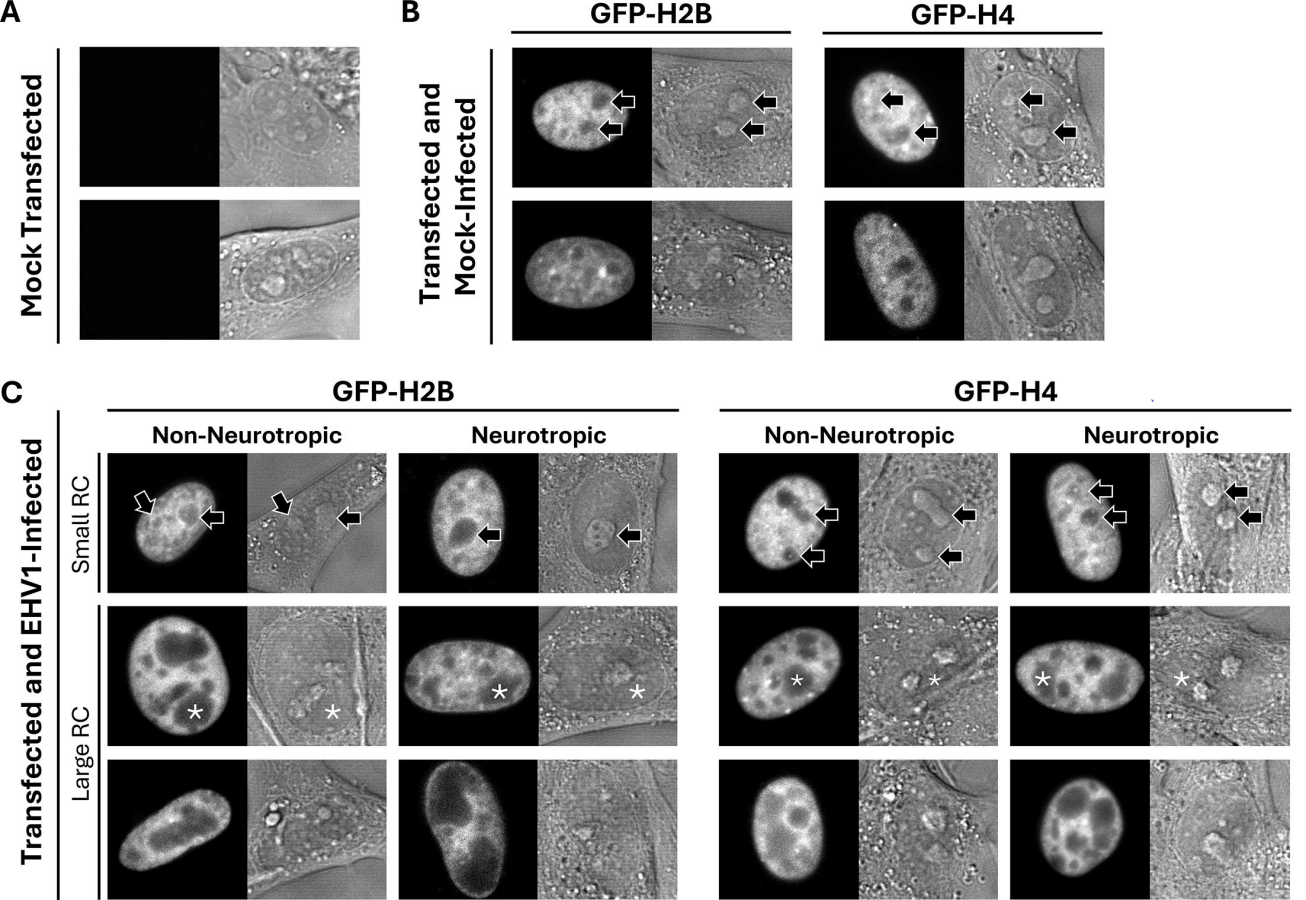
GFP-H2B or -H4 is nuclear and depleted from nucleoli and EHV1 RCs. Digital fluorescent (left panels) and differential interference contrast (DIC; right panels) micrographs show the nucleus of EDerm cells not expressing any GFP-histones (**A**) or expressing GFP fused to H2B or H4 (**B, C**). Cells were mock-transfected (**A**) or transfected with plasmids encoding GFP-H2B or -H4 (**B, C**). At least 40 h after transfection, the cells were mock-infected (**B**) or infected with 10 PFU/cell of non-neurotropic or neurotropic EHV1 (**C**). Live cells were imaged between 5 and 6 hpi. Black arrows (top row in B and C) indicate nucleoli, which are visible as GFP-depleted regions in fluorescent images and as discrete domains in DIC images of mock- or EHV1-infected cells. Note that nucleoli remain during EHV1 infection. White asterisks (middle row in C) mark examples of EHV1 RCs, which are visible as GFP-depleted regions in fluorescent images and are not visible in DIC images. Note that only one RC per cell is marked for clarity. Fluorescent images presented in grayscale for visibility.

For FRAP analyses, equine cells were transiently transfected with plasmids expressing green fluorescent protein (GFP) fused to the N-terminus of equine histones. Mock-transfected equine cells did not have nuclear fluorescence (Fig. 2A), whereas cells transfected with GFP-H2B or -H4 did (Fig. 2B). Transiently expressed GFP-H2B or -H4 was nuclear and had distinct granular localization with relatively enriched or depleted regions consistent with their assembly in chromatin (Fig. 2B). Most cells had nucleoli depleted for GFP-H2B or -H4, which is consistent with nucleolar chromatin instability (Fig. 2B, nucleoli marked by arrows in top row) (38, 82, 83). In infected cells, non-neurotropic or neurotropic EHV1 RCs were evident as domains generally depleted for GFP-H2B or -H4 (Fig. 2C, marked by white asterisks in middle row). In contrast to nucleoli, EHV1 RCs are not identifiable as discrete domains in DIC images (Fig. 2C compare nucleoli marked by arrows in top row to RCs marked by asterisks in middle row). As expected for field-isolated strains, infection progression (evaluated by RC number and size) was variable. Most cells had GFP-H2B- or -H4-depleted regions clearly identifiable as RCs (Fig. 2C, large RC; examples marked by white asterisks in middle row; Table S1). However, smaller populations of cells had GFP-H2B or -H4-depleted regions that resembled those in mock-infected cells (Fig. 2C, small RC). Depletion of GFP-H2B or -H4 from EHV1 RCs is consistent with the reported depletion of histones from HSV1 RCs (55, 76). It is notable that nucleoli were not visibly disrupted during EHV1 infection of equine cells (Fig. 2C). They remained as discrete domains regardless of the number or size of EHV1 RCs (Fig. 2C, nucleoli marked by arrows in top row).

**Fig 3:**
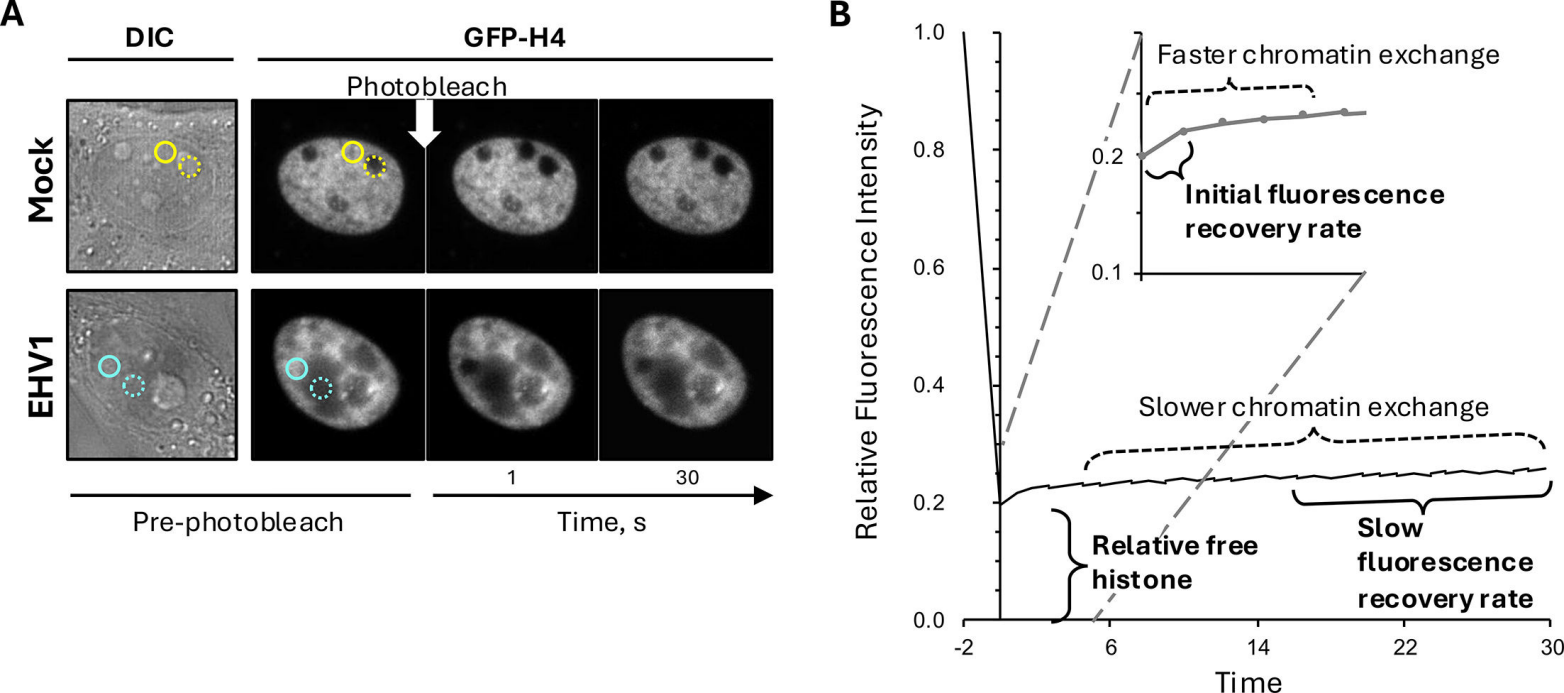
Representative FRAP of GFP-core histones. EDerm cells transfected with plasmids expressing GFP-H4 were mock-infected or infected with 10 PFU/cell of EHV1 at least 40 h after transfection. GFP-H4 mobility was evaluated between 5 and 6 hpi. (**A**) Digital DIC (left panel) and fluorescent (right panels) micrographs of the nucleus of cells expressing GFP-H4 before and at 1 or 30 s after photobleaching. Equal volume regions within the cell chromatin and a nucleolus of the same mock-infected cell (yellow solid and dashed circles, respectively) or the infected-cell chromatin and a RC of the same EHV1-infected cell (cyan solid and dashed circles, respectively) were selected for photobleaching. Fluorescence recovery within these regions, which occurs as photobleached GFP-H4 within them exchanges with fluorescent GFP-H4 outside them, was measured over time. Fluorescent images presented in grayscale for visibility. (**B**) Line graph of a representative GFP-H4 FRAP within mock-infected cell chromatin (an area marked by solid yellow circle in A). Fluorescence intensity of the photobleached region at each time is normalized to the fluorescence intensity of the entire nucleus at that same time. It is then expressed relative to the normalized fluorescence intensity of the same photobleached region before photobleaching and plotted against time after photobleaching. FRAP is therefore independent of GFP-histone expression levels within any given cell. The first data point after photobleaching is set at 0 s due to time differences to photobleach two regions within the same nucleus. The first data point after photobleaching is a surrogate measure for the level of free GFP-histone as only freely diffusing histones can move into photobleached regions in this time. Subsequent fluorescence recovery is biphasic. An initial faster phase represents histones weakly bound in chromatin and undergoing fast chromatin exchange. As a surrogate measure for this population, we calculated the initial rate of normalized fluorescence recovery (slope between normalized fluorescence at the first and second data points after photobleaching; shown in inset). The second slower phase of fluorescence recovery represents histones more stably bound in chromatin and undergoing slow chromatin exchange. As a surrogate measure for this population, we calculated the slow rate of fluorescence recovery (slope between normalized fluorescence at 15 and 30 s data points after photobleaching). Histones available in the free pool or undergoing fast chromatin exchange are most likely available to assemble in EHV1 chromatin in a timescale relevant to lytic infection.

We evaluated histone mobility between 5 and 6 h after infection when most cells had identifiable RCs of various sizes (for examples, see Fig. 2C; Table S1). Thus, we evaluated histone mobility in a heterogeneous population of infected cells with various levels of transcription, IE (immediate early), E (early), or L (late) protein expression, and DNA replication. Histone mobility within a single EHV1 RC and the surrounding infected-cell chromatin was measured in the same cell (Fig. 3A; regions marked by cyan-dashed and solid circles, respectively). We could thus comparatively evaluate histone mobility within areas enriched for EHV1- or cellular-chromatin (Fig. 3A). EHV1 RCs are highly transcriptionally active domains. Therefore, histone mobility within an individual mock-infected nucleoli was measured as a comparator for histone mobility within a highly transcriptionally active region (Fig. 3A; region marked by yellow dashed circle). Equal volume areas were selected for photobleaching within a mock-infected nucleoli (identified by DIC imaging) and the surrounding cell chromatin of the same cell, or within an EHV1 RC (identified by GFP depletion by fluorescent imaging) and the surrounding cell chromatin of the same infected cell (Fig. 3A). Fluorescence recovery within the photobleached regions, which represents GFP-histone mobility, was then measured over time. Most core histones typically stably assemble in chromatin, and their exchange occurs in the magnitude of hours (55–57, 63, 64, 76, 84, 85). This histone population is unlikely to undergo chromatin exchange in a timescale relevant for assembly in lytic EHV1 chromatin. Smaller populations of core histone are, however, at any given time not assembled in chromatin and available in the free histone pool or more weakly bound in chromatin and undergoing fast exchange (Fig. 3B). We therefore measured histone mobility with a focus on the dynamic histone populations most likely available to assemble in lytic EHV1 chromatin (Fig. 3B).

**Fig 4:**
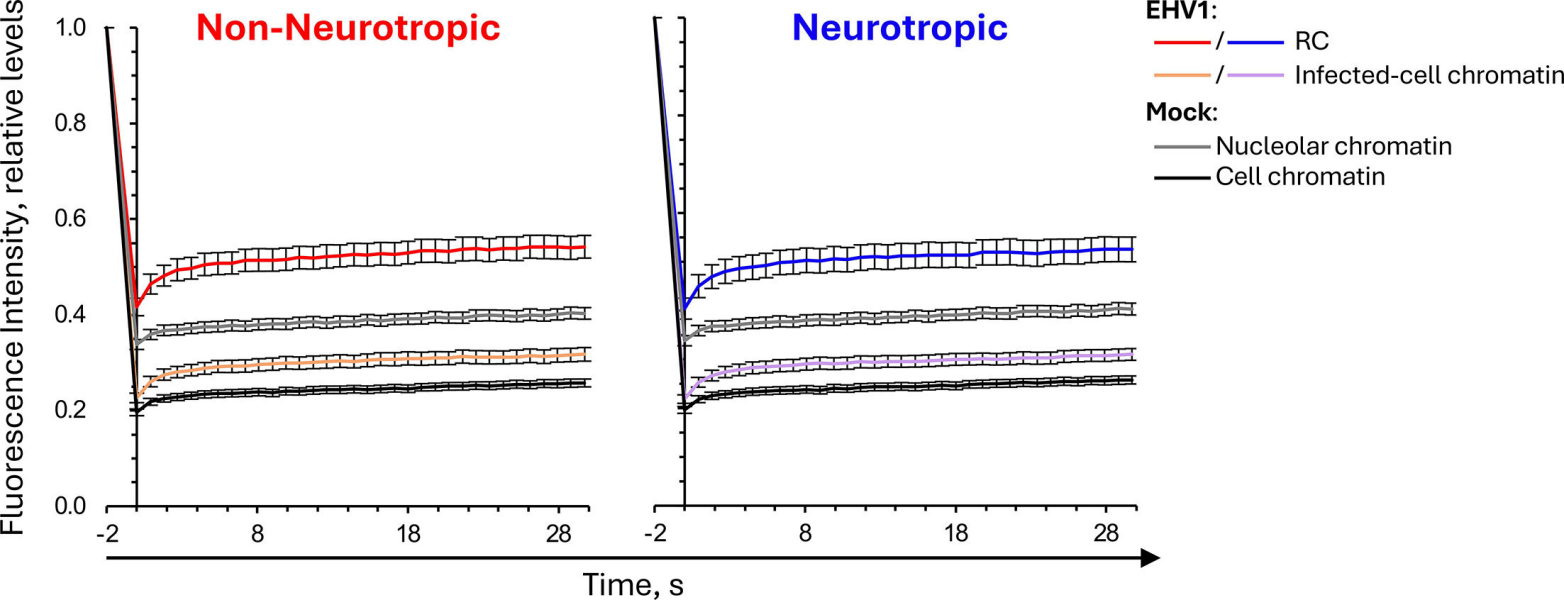
GFP-H4 fluorescence recovers faster in EHV1-infected than in mock-infected cells. EDerm cells transfected with plasmids encoding GFP-H4 were mock-infected or infected with 10 PFU/cell of non-neurotropic or neurotropic EHV1 at least 40 h after transfection. GFP-H4 mobility was evaluated between 5 and 6 hpi by FRAP. Line graphs present the normalized fluorescence intensities of the photobleached nuclear regions plotted against time after photobleaching. FRAP of GFP-H4 in mock-infected cell or nucleolar chromatin plotted in both graphs for comparison. Error bars, standard errors of the means (SEM); *n* ≥ 39 cells per treatment from four independent experiments.

GFP-H4 fluorescence recovered faster within mock-infected nucleoli than within cellular chromatin (Fig. 4; for example, see Fig. 3B regions marked by yellow dashed and solid circles, respectively). Thus, as expected, equine GFP-H4 was more mobile within nucleoli where highly transcribed and unstable nucleolar chromatin is located. Infection with non-neurotropic or neurotropic EHV1 mobilized GFP-H4 such that fluorescence recovered faster in infected cells (Fig. 4). GFP-H4 fluorescence recovered even faster within EHV1 RCs than within the surrounding infected-cell chromatin (Fig. 4; for example, see Fig. 3B regions marked by cyan dashed and solid circles, respectively). These data show that EHV1 infection mobilized H4 and mobilized it to a greater degree in RCs where EHV1 chromatin is enriched. H4 was more mobile within RCs than within mock-infected nucleoli.

GFP-H2B or -H4 were similarly mobilized by non-neurotropic or neurotropic EHV1 (Fig. 4; data not shown). Given the heterogeneity of infection with field-isolated strains, we considered that levels of EHV1 transcription, protein expression, or DNA replication may directly or indirectly affect H2B or H4 mobility. We therefore pooled the infected cell FRAP data for each histone and segregated it by the presence or absence of clearly visually identifiable RCs rather than by infecting strain. Cells with GFP-H2B or -H4-depleted regions visually identifiable as RCs were grouped as “large” RCs, whereas cells without visually identifiable RCs were grouped as “small” RCs (for examples see Fig. 2C). In total, 76% or 70% of GFP-H2B expressing cells or 77% or 67% of GFP-H4 expressing ones infected with non-neurotropic or neurotropic EHV1, respectively, had identifiable “large” RCs (Table S1).

**Fig 5:**
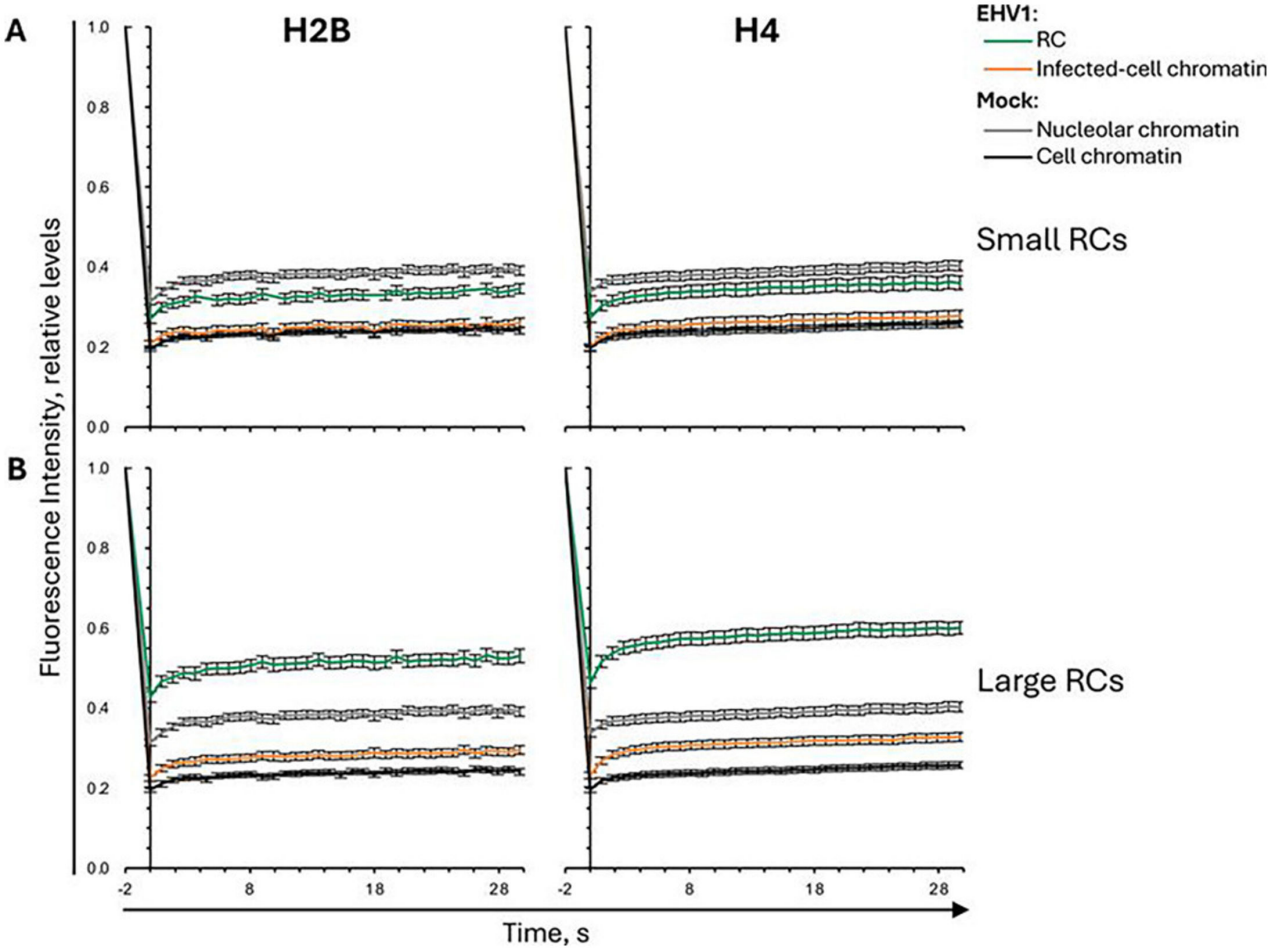
H2B or H4 are most mobile within RCs. EDerm cells transfected with plasmids encoding GFP-H2B or -H4 were mock-infected or infected with 10 PFU/cell of non-neurotropic or neurotropic EHV1 at least 40 h after transfection. GFP-H2B or -H4 nuclear mobility was evaluated by FRAP between 5 and 6 hpi. FRAP data for EHV1-infected cells were pooled for each histone and segregated by the absence (**A**) or presence (**B**) of clearly visually identifiable RCs (for examples, see Fig. 2C). Line graphs present GFP-H2B or -H4 FRAP in RCs or infected-cell chromatin of EHV1-infected cells, or nucleolar or cell chromatin of mock-infected cells. GFP-H4 FRAP data presented in Fig. 3 are re-analyzed and re-plotted. FRAP of GFP-H2B or -H4 in mock-infected cells plotted in both graphs for comparison. Error bars, SEM; n ≥ 38 cells per treatment from four independent experiments.

GFP-H2B or -H4 fluorescence recovery was still enhanced within “small” RCs, although it recovered slower than within mock-infected nucleoli (Fig. 5A). Fluorescence recovery in the surrounding infected-cell chromatin was similar to that in mock-infected cell chromatin (Fig. 5A). GFP-H2B or -H4 was much more mobile within “large” RCs, where fluorescence recovered even faster than within mock-infected nucleoli (Fig. 5B). Mobilization of H2B or H4 in cells with “large” RCs also increased their mobility within the surrounding infected-cell chromatin (Fig. 5B).

These data show that EHV1 mobilized equine H2B and H4 in equine cells. Histones were most mobile within RCs, illustrating that H2B and H4 are most dynamic in domains enriched for EHV1 chromatin. H2B and H4 were mobilized to a greater degree in RCs when infection was further progressed. Therefore, increased levels of EHV1 transcription; IE, E, or L protein expression; or DNA replication directly or indirectly enhance H2B and H4 mobility. Within “large” RCs, H2B and H4 were more mobile than within mock-infected nucleoli, suggesting that EHV1 chromatin is more dynamic than nucleolar chromatin.

### EHV1 mobilizes the most dynamic H2B and H4 populations

**Fig 6:**
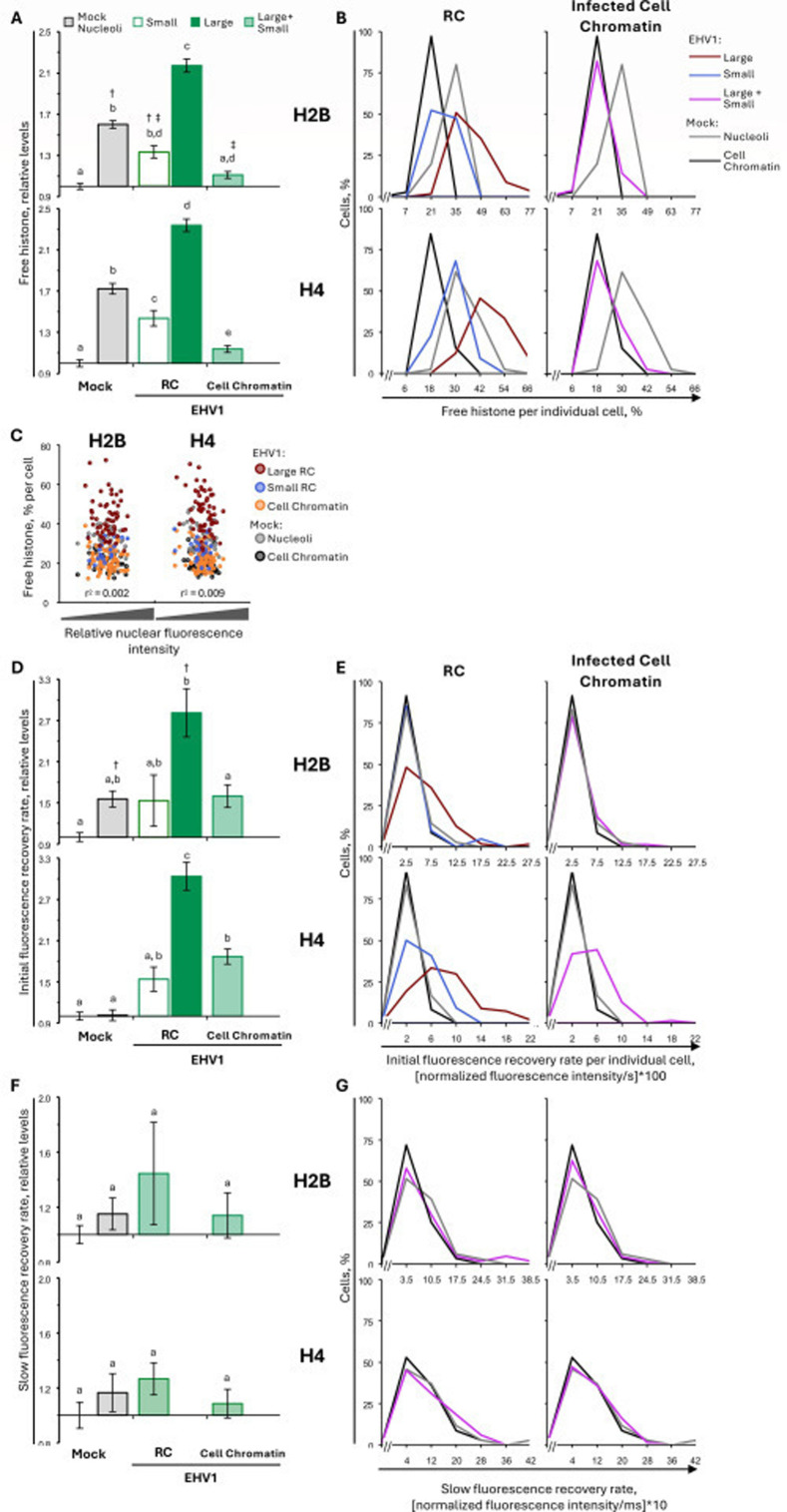
EHV1 mobilizes unbound and weakly bound H2B or H4 within RCs. EDerm cells transfected with plasmids encoding GFP-H2B or -H4 were mock-infected or infected with 10 PFU/cell of non-neurotropic or neurotropic EHV1 at least 40 h after transfection. GFP-H2B or -H4 nuclear mobility was evaluated by FRAP between 5 and 6 hpi. FRAP data for EHV1-infected cells were pooled for each histone and segregated by the absence (small) or presence (large) of clearly identifiable RCs. (**A**) Bar graphs present the average normalized GFP-H2B or -H4 free pool expressed relative to their average normalized free pool in mock-infected cell chromatin (set at 1). (**B**) Frequency distribution plots show the percentage of free GFP-H2B or -H4 per individual cell. (**C**) Dot plot presents the GFP-H2B or -H4 free pool per individual cell plotted against its normalized total nuclear fluorescence intensity before photobleaching. (**D**) Bar graphs present the average GFP-H2B or -H4 initial normalized fluorescence recovery expressed relative to their average initial normalized fluorescence recovery in mock-infected cell chromatin (set at 1). (**E**) Frequency distribution plots present the GFP-H2B or -H4 initial normalized fluorescence recovery per individual cell. (**F**) Bar graphs present the average GFP-H2B or -H4 slow normalized fluorescence recovery expressed relative to their average slow normalized fluorescence recovery in mock-infected cell chromatin (set at 1). (**G**) Frequency distribution plots show the GFP-H2B or -H4 slow normalized fluorescence recovery per individual cell. (**B, E, G**) Data for mock-infected cells plotted in both graphs for comparison. Error bars, SEM. n ≥ 38 cells per treatment from four independent experiments. Different letters denote *P* < 0.01; matching symbols denote *P* < 0.05. Statistical significance evaluated by ANOVA with post-hoc Tukey Kramer pair-wise analysis.

To investigate the H2B and H4 populations mobilized by EHV1, we examined GFP-H2B and -H4 fluorescence recovery kinetics. As a surrogate measure for histones in the free pool (not assembled in chromatin and diffusing in the nucleoplasm), we used the first value after photobleaching (Fig. 3B). Only freely diffusing histones enter photobleached regions within this time. In mock-infected cells, nucleolar H2B or H4 free pools were 160% ± 4% or 172% ± 5%, respectively, of their levels within the surrounding cellular chromatin, consistent with the dynamic instability of nucleolar chromatin (*P* < 0.01; Fig. 6A; Table 3). H2B or H4 free pools tended to increase within the infected-cell chromatin, to 111% ± 3% or 114% ± 3%, respectively, of their levels within mock-infected cell chromatin (*P* = ns or <0.01, respectively; Fig. 6A; Table 3). However, H2B or H4 free pools were larger in domains enriched for viral chromatin. In “small” RCs, free H2B or H4 levels increased to 133% ± 6% or 144% ± 8%, respectively, relative to mock-infected cell chromatin (*P* < 0.01; Fig. 6A; Table 3). Free H2B or H4 pools even further increased within “large” RCs, to 217% ± 6% or 234% ± 6%, respectively (*P* < 0.01; Fig. 6A; Table 3). The levels of free H2B or H4 within “large” RCs were significantly greater than within mock-infected nucleoli, further supporting that H2B and H4 are most dynamic within “large” RCs (*P* < 0.01; Fig. 6A; Table 3).

**TABLE 3:** Free histone levels^*d*^

			Level of free histone (avg ± SEM)		P	Cells with large increase^*a*^ (%)
			Absolute (normalized fluorescence intensity, %)	Relative (% mock cell chromatin)		
H2B	Mock	Chromatin	19.8 ± 0.7	100 ± 3	ns	23
		Nucleolus	31.7 ± 0.8	160 ± 4	**	94
	EHV	Small RC	27.0 ± 1.1	133 ± 6	**	67
		Large RC	42.8 ± 1.3	217 ± 6	**	100
		Cell chromatin	22.0 ± 0.6	111 ± 3	ns	36
H4	Mock	Chromatin	19.6 ± 0.7	100 ± 3	ns	15
		Nucleolus	33.8 ± 1.0	172 ± 5	**	97
	EHV	Small RC	27.3 ± 1.2	144 ± 8	**	77
		Large RC	46.3 ± 1.3	234 ± 6	**	100
		Cell chromatin	22.2 ± 0.7	114 ± 3	**	32
H3.1	Mock	Chromatin	25.1 ± 1.2	100 ± 4	ns	20
		Nucleolus	35.9 ± 1.5	144 ± 5	**	59
	EHV	Small RC	31.2 ± 1.4	117 ± 4	ns	33
		Large RC	43.2 ± 1.5	175 ± 7	**	81
		Cell chromatin	25.4 ± 1.0	101 ± 4	ns	19
		Diffuse	52.3 ± 2.2	256 ± 12	**	100
H3.3	Mock	Chromatin	21.4 ± 1.1	100 ± 5	ns	17
		Nucleolus	37.0 ± 1.3	176 ± 6	**	71
	EHV	Small RC	24.6 ± 1.1	119 ± 7	ns	19
		Large RC	48.6 ± 1.3	237 ± 7	**	99
		Small cell chromatin	18.7 ± 1.2	91 ± 7	ns	8^*b*^
		Large cell chromatin	26.6 ± 1.0	129 ± 5	*	22
		Diffuse	58.4 ± 1.4	270 ± 9	**	98
H2A	Mock	Chromatin	20.2 ± 0.7	100 ± 3	ns	20
		Nucleolus	31.1 ± 1.0	155 ± 5	**	83
	EHV	Small RC	25.8 ± 1.3	127 ± 6	*	55
		Large RC	47.7 ± 1.1	238 ± 5	**	100
		Cell chromatin	21.6 ± 0.6	107 ± 3	ns	29
H2A.Z	Mock	Chromatin	23.3 ± 0.6	100 ± 3	ns	10
		Nucleolus	33.4 ± 1.0	143 ± 4	**	90
	EHV	Small RC	33.8 ± 2.0	147 ± 8	**	83
		Large RC	50.3 ± 1.1	215 ± 5	**	100
		Cell chromatin	24.2 ± 0.7	104 ± 3	ns	30
H2A.X	Mock	Chromatin	20.6 ± 0.7	100 ± 3	ns	20
		Nucleolus	33.1 ± 1.2	160 ± 5	**	90
	EHV	Small RC	34.4 ± 1.8	169 ± 9	**	93
		Large RC	47.2 ± 1.2	229 ± 6	**	100
		Cell chromatin	21.5 ± 0.6	104 ± 3	ns	24
MacroH2A	Mock	Chromatin	18.3v ± 0.7	100 ± 3	ns	13
		Nucleolus	29.4 ± 0.9	161 ± 5	**	93
	EHV	Small RC	26.0 ± 1.1	147 ± 8	**	74
		Large RC	42.6 ± 1.1	233 ± 6	**	100
		Cell chromatin	20.1 ± 0.6	111 ± 4	ns	30
H2A.B^*c*^	Mock	Nucleolus	23.0 ± 0.7	67 ± 2	**	92^*b*^
		Chromatin 1	23.3 ± 2.1	67 ± 6	*	100^*b*^
		Chromatin 2	28.6 ± 0.9	83 ± 3	ns	50^*b*^
		Chromatin 3	34.5 ± 1.3	100 ± 4	ns	7
		Chromatin 4	39.1 ± 1.5	113 ± 4	ns	57
	EHV	Chromatin 1	25.1 ± 1.7	73 ± 5	**	80^*b*^
		Chromatin 2	32.2 ± 1.0	93 ± 3	ns	37^*b*^
		Chromatin 3	32.1 ± 0.8	93 ± 2	ns	25^*b*^
		Chromatin 4+	51.5 ± 1.1	149 ± 3	**	94
H1.2	Mock	Chromatin	23.2 ± 0.6	100 ± 2	ns	21
		Nucleolus	27.4 ± 0.7	119 ± 3	*	44
	EHV	Small RC	31.5 ± 1.6	137 ± 6	**	68
		Large RC	44.0 ± 1.3	191 ± 6	**	100
		Cell chromatin	22.2 ± 0.6	97 ± 3	ns	18

^*a*^ Percentage of cells > 1 SD above mock-infected cell chromatin average.

^*b*^ Percentage of cells > 1 SD below mock-infected cell chromatin average.

^*c*^ H2A.B normalized to mobility group 3 in mock-infected cell chromatin.

^*d*^ *P* values represent Tukey Kramer post-hoc pairwise comparison with mock-infected cell chromatin. **P < 0.01, *P <0.05, ns, not significantly different.

As it was possible that a subpopulation of infected cells increased their H2B or H4 free pools by an extreme degree while others had little or no change, we evaluated the level of free H2B or H4 per individual cell. The frequency distribution of H2B or H4 free pool levels within the cell chromatin of every EHV1- or mock-infected cell was unimodal and largely overlapped, consistent with their similar average free pool levels (Fig. 6B; plotted as lines for clarity and comparative analysis of overlayed populations). The frequency distribution of free H2B or H4 within a “small” or “large” RC per individual cell was also unimodal, indicating that H2B and H4 free pools increased within RCs throughout the infected cell population (Fig. 6B). Consistent with the average increase in H2B or H4 free pools within “small” or “large” RCs, their frequency distributions shifted to the right relative to mock-infected cell chromatin (Fig. 6B). Most “small” RCs had free H2B or H4 levels greater than one standard deviation (SD) above their average level within mock-infected cell chromatin (67% or 77% of cells, respectively; Table 3). The free pools of H2B or H4 increased by a large degree (>1 SD above the mock-infected cell chromatin average) in all “large” RCs (Table 3). Furthermore, 70% or 68% of cells had H2B or H4 free pools within “large” RCs greater than 1 SD above their average level in mock-infected nucleoli.

To ensure that H2B or H4 free pools were not increased due to GFP-H2B or -H4 expression levels, the level of free histone per individual cell was plotted relative to its total nuclear fluorescence intensity before photobleaching (Fig. 6C). Free H2B or H4 levels in any evaluated nuclear domain of individual mock- or EHV1-infected cells did not correlate with total nuclear fluorescence. Thus, H2B or H4 free pools within EHV1, nucleolar, or cellular chromatin were independent of the overall GFP-histone expression level in any given cell.

To identify whether EHV1 affected H2B or H4 fast chromatin exchange, we next evaluated their initial normalized fluorescence recovery rates (Fig. 3B). H2B or H4 fast chromatin exchange tended to increase within “small” RCs (to 153% ± 37% or 154% ± 18%, respectively), although these rates were not significantly different from those within mock-infected cell chromatin (Fig. 6D; Table 4). Nonetheless, 30% or 55% of cells had a large increase in H2B or H4 fast chromatin exchange within “small” RCs (>1 SD higher than the mock-infected cell chromatin average), which is larger than expected in a normal population were they not mobilized (Fig. 6E; Table 4). The fast chromatin exchange of H2B or H4 further increased within “large” RCs (to 281% ± 35% or 304% ± 20%, respectively; *P* < 0.01; Fig. 6D; Table 4). H2B or H4 fast chromatin exchange increased within “large” RCs throughout the infected cell population, with a large increase (>1 SD above the mock-infected cell chromatin average) in 56% or 84% of cells, respectively (Fig. 6E; Table 4). H4 mobilization within EHV1-infected cells also affected its fast chromatin exchange within the surrounding infected-cell chromatin, where it increased to 187 ± 11% (*P* < 0.01; Fig. 6D and E; Table 4).

**TABLE 4:** Initial normalized fluorescence recovery rates^*d*^

			Initial fluorescence recovery rates (avg ± SEM)		*P*	Cells with large increase^*a*^ (%)
			Absolute (normalized fluorescence intensity/ms)	Relative (% mock cell chromatin)		
H2B	Mock	Chromatin	27.5 ± 2.5	100 ± 7	ns	11
		Nucleolus	42.3 ± 4.0	156 ± 12	ns	29
	EHV	Small RC	41.3 ± 6.4	153 ± 37	ns	30
		Large RC	61.9 ± 61.9	281 ± 35	**	56
		Cell chromatin	36.7 ± 2.9	160 ± 16	ns	28
H4	Mock	Chromatin	27.5 ± 1.7	100 ± 6	ns	16
		Nucleolus	27.9 ± 2.2	101 ± 8	ns	19
	EHV	Small RC	40.8 ± 4.5	154 ± 18	ns	55
		Large RC	84.5 ± 6.0	304 ± 20	**	84
		Cell chromatin	51.4 ± 3.3	187 ± 11	**	65
H3.1	Mock	Chromatin	73.9 ± 8.9	100 ± 10	ns	13
		Nucleolus	84.0 ± 10.5	110 ± 10	ns	15
	EHV	Small RC	75.7 ± 12.3	85 ± 15	ns	7
		Large RC	90.0 ± 8.2	155 ± 18	ns	25
		Cell chromatin	61.8 ± 6.2	94 ± 10	ns	12
		Diffuse	215.3 ± 19.1	373 ± 36	**	82
H3.3	Mock	Chromatin	44.3 ± 7.1	100 ± 11	ns	10
		Nucleolus	46.6 ± 5.5	118 ± 14	ns	14
	EHV	Small RC	39.7 ± 6.4	162 ± 39	ns	13
		Large RC	75.7 ± 7.7	260 ± 40	*	23
		Cell chromatin	49.8 ± 4.6	157 ± 18	ns	11
		Diffuse	194.7 ± 11.5	502 ± 40	**	89
H2A	Mock	Chromatin	24.3 ± 2.0	100 ± 5	ns	11
		Nucleolus	37.3 ± 2.9	162 ± 10	ns	36
	EHV	Small RC	31.1 ± 4.6	110 ± 20	ns	32
		Large RC	72.9 ± 5.6	357 ± 27	**	82
		Small cell chromatin	27.6 ± 2.4	99 ± 17	ns	25
		Large cell chromatin	40.8 ± 2.2	202 ± 12	*	44
H2A.Z	Mock	Chromatin	35.0 ± 2.9	100 ± 8	ns	18
		Nucleolus	37.7 ± 2.8	111 ± 9	ns	13
	EHV	Small RC	53.5 ± 8.2	154 ± 28	ns	45
		Large RC	85.0 ± 6.8	263 ± 25	**	70
		Cell chromatin	49.6 ± 3.4	152 ± 13	ns	32
H2A.X	Mock	Chromatin	24.9 ± 1.8	100 ± 6	ns	15
		Nucleolus	34.5 ± 2.8	137 ± 10	ns	43
	EHV	Small RC	42.1 ± 8.9	189 ± 49	ns	36
		Large RC	48.4 ± 4.1	212 ± 21	**	58
		Cell chromatin	31.8 ± 1.8	136 ± 9	ns	25
MacroH2A	Mock	Chromatin	23.1 ± 2.4	100 ± 9	ns	10
		Nucleolus	28.2 ± 2.6	123 ± 11	ns	16
	EHV	Small RC	26.8 ± 2.5	119 ± 13	ns	8
		Large RC	46.9 ± 5.7	210 ± 26	**	48
		Cell chromatin	26.3 ± 1.8	116 ± 9	ns	20
H2A.B^*c*^	Mock	Nucleolus	211.0 ± 10.9	97 ± 5	ns	15
		Chromatin 1	90.3 ± 13.0	42 ± 6	**	100^*b*^
		Chromatin 2	176.9 ± 20.0	81 ± 9	ns	33^*b*^
		Chromatin 3	217.3 ± 20.2	100 ± 9	ns	13
		Chromatin 4	350.6 ± 31.8	161 ± 15	**	71
	EHV	Chromatin 1	85.0 ± 7.1	39 ± 3	**	100^*b*^
		Chromatin 2	130.1 ± 6.0	60 ± 3	**	65^*b*^
		Chromatin 3	132.3 ± 6.1	61 ± 3	**	66^*b*^
		Chromatin 4+	187.5 ± 6.1	86 ± 3	ns	8^*b*^
H1.2	Mock	Chromatin	42.0 ± 1.6	100 ± 4	ns	18
		Nucleolus	41.4 ± 2.4	99 ± 6	ns	13
	EHV	Small RC	59.6 ± 3.7	142 ± 9	**	60
		Large RC	57.5 ± 4.5	138 ± 11	**	57
		Cell chromatin	44.0 ± 1.7	106 ± 4	ns	25

^*a*^ Percentage of cells > 1 SD above mock-infected cell chromatin average.

^*b*^ Percentage of cells > 1 SD below mock-infected cell chromatin average.

^*c*^ H2A.B normalized to mobility group 3 in mock-infected cell chromatin.

^*d*^ *P* values represent Tukey Kramer post-hoc pairwise comparison with mock-infected cell chromatin. **P <0.01, *P <0.05, ns = not significantly different.

To investigate whether EHV1 also mobilized histones more stably assembled in chromatin, we next evaluated H2B and H4 slow normalized fluorescence recovery rates (Fig. 3B). The stable chromatin interactions of H2B and H4 were largely unaffected during infection. H2B or H4 slow chromatin exchange tended to increase within RCs (to 144% ± 16% or 127% ± 12%, respectively); however, this was not significantly different from within mock-infected cell chromatin (Fig. 6F and G; Table 5).

**TABLE 5:** Normalized slow fluorescence recovery rates^*c*^

			Slow fluorescence recovery rates (avg ± SEM)		*P*	Cells with large increase^*a*^ (%)
			Absolute (normalized fluorescence intensity/ ms)	Relative (% mock chromatin)		
H2B	Mock	Chromatin	0.60 ± 0.06	100 ± 10	ns	16
		Nucleolus	0.70 ± 0.09	115 ± 15	ns	15
	EHV	RC	0.88 ± 0.10	144 ± 16	ns	28
		Cell chromatin	0.69 ± 0.05	144 ± 9	ns	24
H4	Mock	Chromatin	0.97 ± 0.10	100 ± 10	ns	17
		Nucleolus	1.09 ± 0.15	116 ± 14	ns	20
	EHV	RC	1.11 ± 0.09	127 ± 12	ns	27
		Cell chromatin	0.95 ± 0.08	108 ± 10	ns	20
H3.1	Mock	Chromatin	1.52 ± 0.31	100 ± 16	ns	13
		Nucleolus	2.06 ± 0.43	130 ± 24	ns	14
	EHV	RC	1.31 ± 0.17	120 ± 23	ns	4
		Cell chromatin	0.88 ± 0.10	77 ± 11	ns	0^*b*^
		Diffuse	1.96 ± 0.28	155 ± 46	ns	14
H3.3	Mock	Chromatin	1.14 ± 0.13	100 ± 10	ns	21
		Nucleolus	1.83 ± 0.20	167 ± 18	ns	39
	EHV	RC	1.66 ± 0.22	168 ± 25	ns	30
		Cell chromatin	1.13 ± 0.12	108 ± 12	ns	11
		Diffuse	1.26 ± 0.17	105 ± 14	ns	13
H2A	Mock	Chromatin	0.71 ± 0.07	100 ± 9	ns	16
		Nucleolus	0.82 ± 0.09	119 ± 13	ns	20
	EHV	RC	1.08 ± 0.09	152 ± 12	*	33
		Cell chromatin	0.90 ± 0.08	126 ± 10	ns	30
H2A.Z	Mock	Chromatin	0.83 ± 0.07	100 ± 8	ns	14
		Nucleolus	0.99 ± 0.11	119 ± 13	ns	32
	EHV	RC	1.67 ± 0.17	204 ± 21	**	60
		Cell chromatin	1.17 ± 0.08	139 ± 10	ns	38
H2A.X	Mock	Chromatin	0.71 ± 0.10	100 ± 12	ns	10
		Nucleolus	0.96 ± 0.11	136 ± 14	ns	30
	EHV	RC	1.15 ± 0.12	191 ± 27	ns	35
		Cell chromatin	0.93 ± 0.11	154 ± 24	ns	15
MacroH2A	Mock	Chromatin	0.78 ± 0.11	100 ± 14	ns	10
		Nucleolus	1.14 ± 0.15	147 ± 20	ns	33
	EHV	RC	1.20 ± 0.12	150 ± 14	ns	25
		Cell chromatin	0.77 ± 0.06	100 ± 8	ns	11

^*a*^ Percentage of cells > 1 SD above mock-infected cell chromatin average.

^*b*^ Percentage of cells > 1 SD below mock-infected cell chromatin average.

^*c*^ *P* values represent Tukey Kramer post-hoc pairwise comparison with mock-infected cell chromatin. **P <0.01, *P <0.05, ns = not significantly different.

Together, these data show that EHV1 primarily mobilized the histone populations most likely to assemble in, and undergo exchange with, EHV1 chromatin on a timescale relevant to lytic infection. Mobilization caused a net increase in H2B and H4 free pools and increased their fast chromatin exchange within RCs. H4 was also mobilized within infected-cell chromatin. EHV1 did not significantly mobilize H2B or H4 more stably bound in chromatin and undergoing slow exchange.

### EHV1 mobilizes equine canonical H3.1 and variant H3.3

**Fig 7:**
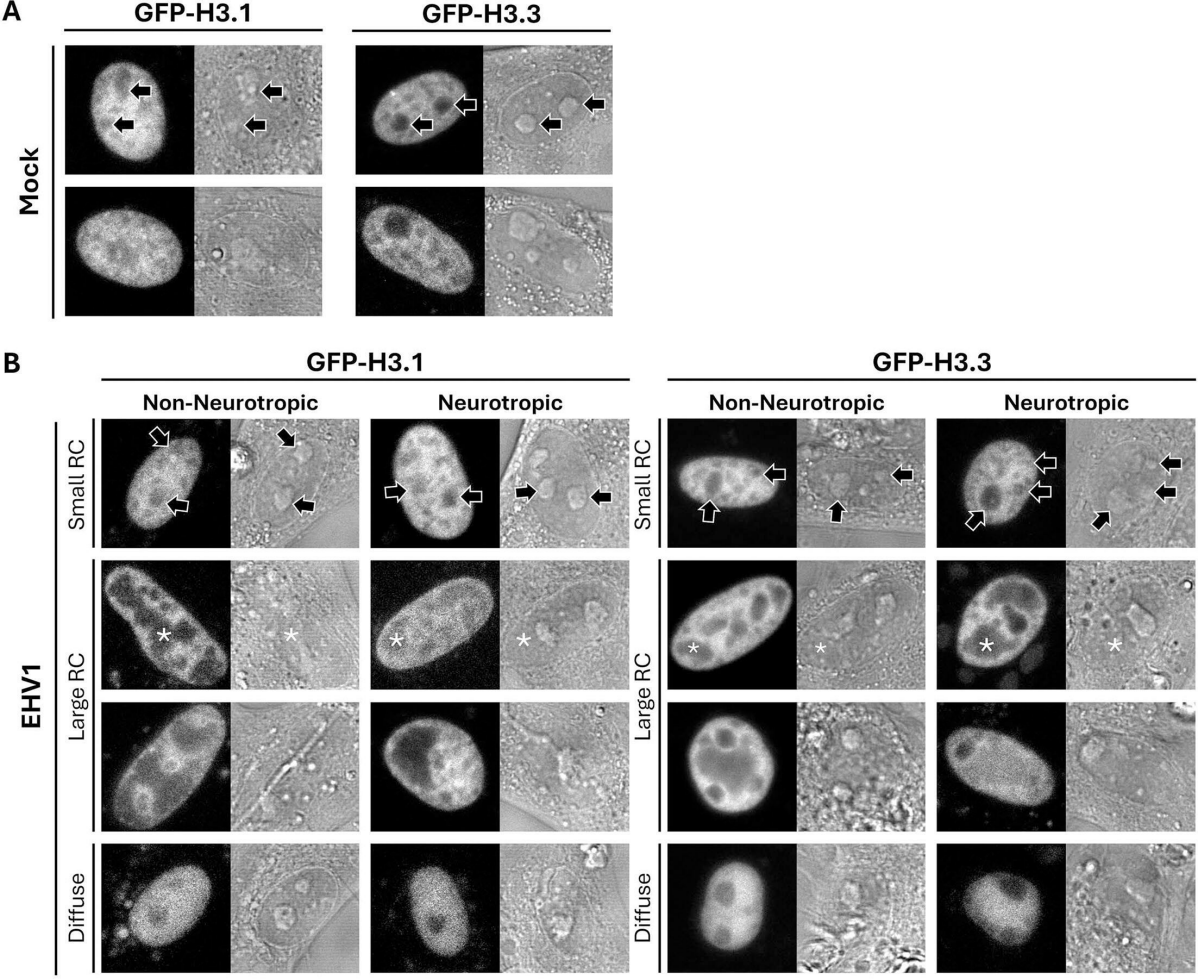
GFP-H3.1 or -H3.3 is variably depleted from nucleoli and RCs. Digital fluorescent (left panels) and DIC (right panels) micrographs show the nucleus of EDerm cells expressing GFP-H3.1 or -H3.3. Cells transfected with plasmids encoding GFP-H3.1 or -H3.3 were mock-infected (**A**) or infected with 10 PFU/cell of non-neurotropic or neurotropic EHV1 (**B**) at least 40 h after transfection. Live cells were imaged between 5 and 6 hpi. Black arrows (top row in **A and B**) indicate nucleoli, which are visible in fluorescent and DIC images of mock- or EHV1-infected cells. White asterisks (second row in **B**) mark examples of EHV1 RCs, which are visible in fluorescent images but not in DIC images. Note that only one RC per cell is marked for clarity. Fluorescent images presented in grayscale for visibility.

We next measured equine canonical H3.1 and variant H3.3 mobility to test whether EHV1 differentially mobilizes particular histone variants (as does HSV1) (56, 57, 76). GFP-H3.1 or -H3.3 had discrete granular nuclear localization consistent with their assembly in chromatin (Fig. 7A). As for GFP-H2B and -H4, GFP-H3.1 and -H3.3 were relatively depleted from nucleoli (Fig. 7A; for examples, see regions marked by arrows in top row). GFP-H3.1 generally appeared more diffuse than GFP-H3.3 due to less pronounced depletion from nucleoli and other domains (Fig. 7A). In most EHV1-infected cells, GFP-H3.1 or -H3.3 was largely depleted from RCs (Fig. 7B; for examples, see regions marked by asterisks in the second row). However, some cells had less pronounced depletion from “large” RCs, and a minor cell subpopulation had diffuse GFP-H3.1 or -H3.3 with relative depletion of either histone only apparent from nucleoli (Fig. 7B Diffuse; Table S1). We first considered that this localization may indicate the lack of GFP-H3.1 or -H3.3 assembly in chromatin. However, we included this cell population in our analyses for several reasons. First, the cells were transfected with GFP-H3.1 or -H3.3 expression constructs at least 40 h prior to infection for FRAP analysis to ensure multiple cell divisions for GFP-histone assembly in chromatin during S-phase. Second, diffuse GFP-histone localization was only observed in infected cells expressing GFP-H3.1 or -H3.3, not in mock-infected cells or cells expressing any other GFP-histone evaluated herein (Fig. 2, 7, 9, 11, and 13). Third, truly freely diffusing GFP-H3.1 or -H3.3 would not be expected to be notably excluded from nucleoli (Fig. 7B). Finally, diffuse GFP-H3.1 or -H3.3 localization is also apparent in cells transiently over-expressing the HSV1 transcription activator ICP4, which is sufficient to mobilize histones (76). We therefore considered that diffuse GFP-H3.1 or -H3.3 may represent a cell subpopulation with extreme EHV1 mobilization of H3 and included these cells in subsequent analyses.

**Fig 8:**
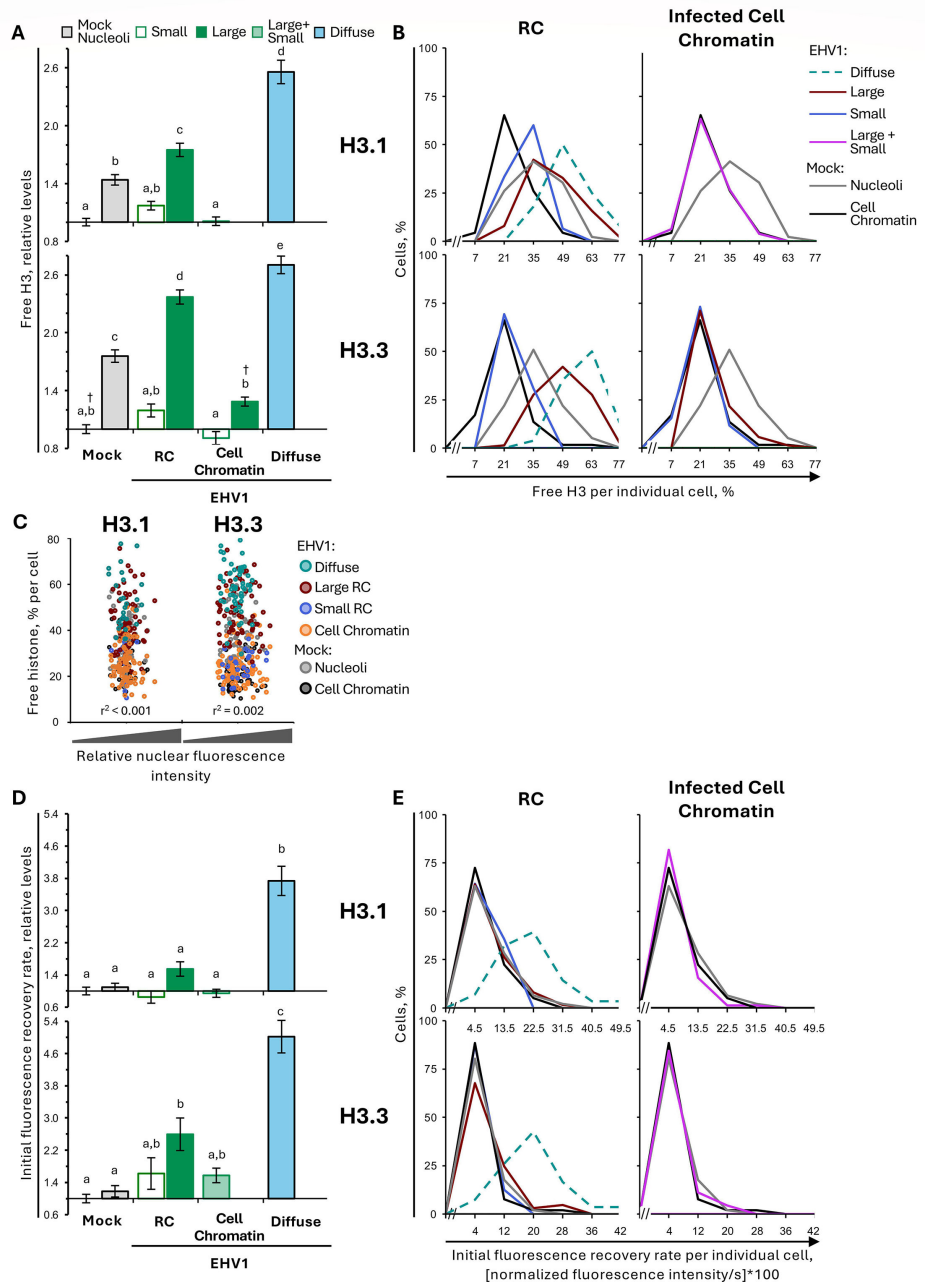
H3.1 and H3.3 are most dynamic within “large” RCs and cells with diffuse H3. EDerm cells transfected with plasmids encoding GFP-H3.1 or -H3.3 were mock-infected or infected with 10 PFU/cell of non-neurotropic or neurotropic EHV1 at least 40 h after transfection. GFP-H3.1 or -H3.3 nuclear mobility was evaluated by FRAP between 5 and 6 hpi. FRAP data for EHV1-infected cells were pooled for each histone and segregated by the absence (small) or presence (large) of clearly identifiable RCs (for examples, see Fig. 7B). (**A**) Bar graphs present the average GFP-H3.1 or -H3.3 normalized free pools relative to their average normalized free pool in mock-infected cell chromatin (set at 1). (**B**) Frequency distribution plots show the percentage of free GFP-H3.1 or -H3.3 per individual cell. (**C**) Dot plot presents GFP-H3.1 or -H3.3 free pools for each cell plotted against its normalized total nuclear fluorescence intensity before photobleaching. (**D**) Bar graphs present the average GFP-H3.1 or -H3.3 initial normalized fluorescence recovery relative to their average initial normalized fluorescence recovery in mock-infected cell chromatin (set at 1). (**E**) Frequency distribution plots present GFP-H3.1 or -H3.3 initial normalized fluorescence recovery per individual cell. (**B, E**) Data for mock-infected cells plotted in both graphs for comparison. Error bars, SEM. H3.1 n ≥ 46 cells per treatment from five independent experiments; H3.3 n ≥ 60 cells per treatment from six independent experiments. Different letters denote *P* < 0.01; matching symbols denote *P* < 0.05. Statistical significance evaluated by ANOVA with post-hoc Tukey Kramer pair-wise analysis.

EHV1 infection mobilized H3.1 and H3.3 within RCs, where EHV1 chromatin is enriched (Fig. S3). Within “large” RCs, H3.1 or H3.3 free pools increased to 175% ± 7% or 237% ± 7%, respectively (*P* < 0.01; Fig. 8A; Table 3). They increased throughout the cell population, with a large increase (>1 SD above the mock-infected cell chromatin average) in 81% or 99% of the cells, respectively (Fig. 8A and B; Table 3). Moreover, H3.1 or H3.3 free pools within “large” RCs were significantly greater than in mock-infected nucleoli (144% ± 5% or 176% ± 6%, respectively; *P* < 0.01), and 34% or 52% of the cells had free pools greater than 1 SD above their average level in mock-infected nucleoli (Fig. 8A and B; Table 3). The high levels of free H3.1 or H3.3 within “large” RCs were not due to GFP-histone expression levels (Fig. 8C).

Although H3.1 and H3.3 were mobilized within “large” RCs, neither histone was significantly mobilized within “small” ones. H3.1 or H3.3 free pools only tended to increase within “small” RCs (to 117% ± 4% or 119% ± 7%, respectively; *P* = ns; Fig. 8A and B; Table 3). Infection also did not substantially mobilize H3.1 or H3.3 within infected-cell chromatin. Only the H3.3 free pool increased to 129% ± 5%, within cell chromatin surrounding “large” RCs (*P* < 0.05; Fig. 8A and B; Table 3).

It was not possible to identify nuclear domains likely to be enriched for EHV1 or cell chromatin when H3.1 or H3.3 had diffuse localization. Therefore, H3.1 or H3.3 mobility within these cells represents their net mobility due to cell- and EHV1-chromatin exchange. H3.1 or H3.3 was nonetheless highly mobilized within these cells. Mobilization increased H3.1 or H3.3 free pools to 256% ± 12% or 270% ± 9%, respectively, which were significantly larger than within “large” RCs (*P* < 0.01; Fig. 8A and B; Table 3). The large increase in H3.1 or H3.3 free pools occurred throughout this cell population and did not correlate with the overall GFP-histone expression levels (Fig. 8B and C).

H3.1 and H3.3 mobilization within “large” RCs and cells with diffuse H3 caused a net increase in their free pools. In cells with diffuse H3.1 or H3.3, this was accompanied by increased fast chromatin exchange (373% ± 36% or 502% ± 40%, respectively, *P* < 0.01; Fig. 8D and E; Table 4). In contrast, only H3.3 fast chromatin exchange increased (to 260% ± 40%; *P* < 0.05), whereas that of H3.1 was not significantly altered (155% ± 18%), within “large” RCs (Fig. 8D and E; Table 4). However, the analysis of H3.3 fast chromatin exchange per individual cell revealed that it only increased within “large” RCs in a minor cell subpopulation (23% of cells had a rate > 1 SD above the mock-infected cell chromatin average; Fig. 8E; Table 4). Thus, most “large” RCs did not have increased H3.3 fast chromatin exchange within them.

In summary, EHV1 did not significantly mobilize H3.1 or H3.3 within “small” RCs or infected-cell chromatin. Only H3.3 was partially mobilized within cell chromatin surrounding “large” RCs. EHV1 did, however, mobilize H3.1 and H3.3 within “large” RCs and cells with diffuse H3 localization. This mobilization caused a net increase in H3.1 and H3.3 free pools. However, mobilization differentially affected their fast chromatin exchange. Although H3.1 and H3.3 fast chromatin exchange increased in cells with diffuse H3, only H3.3 fast chromatin exchange increased in “large” RCs and only in a minor cell subpopulation. Thus, the free pools of H3.1 and H3.3 primarily increased within “large” RCs without substantial change to their fast chromatin exchange. These data show that H3.1 and H3.3 are differentially mobilized in “large” RCs or cells with diffuse H3 and suggest that different mechanisms contribute to increase H3.1 or H3.3 free pools within either.

### H3.3 is mobilized more than H3.1 in EHV1 “large” RCs

H3.3 was apparently mobilized more than H3.1 in “large” RCs and cells with diffuse H3, at least relative to their respective mobilities within mock-infected cell chromatin (Fig. 8A and D). H3.3 may thus be more susceptible to histone mobilizing factors or, alternatively, H3.1 may have higher intrinsic mobility and therefore lower potential magnitude for mobilization. To investigate these possibilities, we evaluated H3.3 mobilization relative to H3.1 (Fig. S3; Table S2). Surprisingly, H3.3 was less mobile than H3.1 in mock-infected cell chromatin. It had a relatively smaller free pool and slower fast chromatin exchange than H3.1 (85% ± 5% or 60% ± 10%, respectively, *P* < 0.05; Fig. S3; Table S2). Within nucleoli, however, H3.1 and H3.3 free pools were similar, despite significantly slower H3.3 fast chromatin exchange (56% ± 7% that of H3.1, *P* < 0.01; Fig. S3; Table S2).

Infection mobilized H3.3 such that its free pools significantly increased relative to those of H3.1 within “large” RCs or cells with diffuse H3 (113% ± 3%, *P* < 0.01 or 112% ± 3%, *P* < 0.05, respectively; Fig. S3; Table S2). The average H3.3 fast chromatin exchange also increased by a greater magnitude than H3.1 in “large” RCs (260% ± 40% vs 155% ± 18%) and cells with diffuse H3 (502% ± 40% vs 373% ± 36%; Fig. 8D and E). However, this mobilization resulted in relatively similar H3.3 and H3.1 fast chromatin exchange rates (Fig. S3; Table S2).

Together, these data show that equine cells have a dynamic H3.1 population that is intrinsically more mobile than H3.3, with a larger free pool and enhanced fast chromatin exchange. EHV1 mobilized H3.3 by a greater magnitude in “large” RCs and increased its fast chromatin exchange by a greater magnitude in cells with diffuse H3.3. This mobilization resulted in relatively larger H3.3 free pools, despite relatively similar H3.3 and H3.1 fast chromatin exchange.

### EHV1 mobilizes equine canonical H2A and variant H2A.Z, H2A.X, and macroH2A

**Fig 9:**
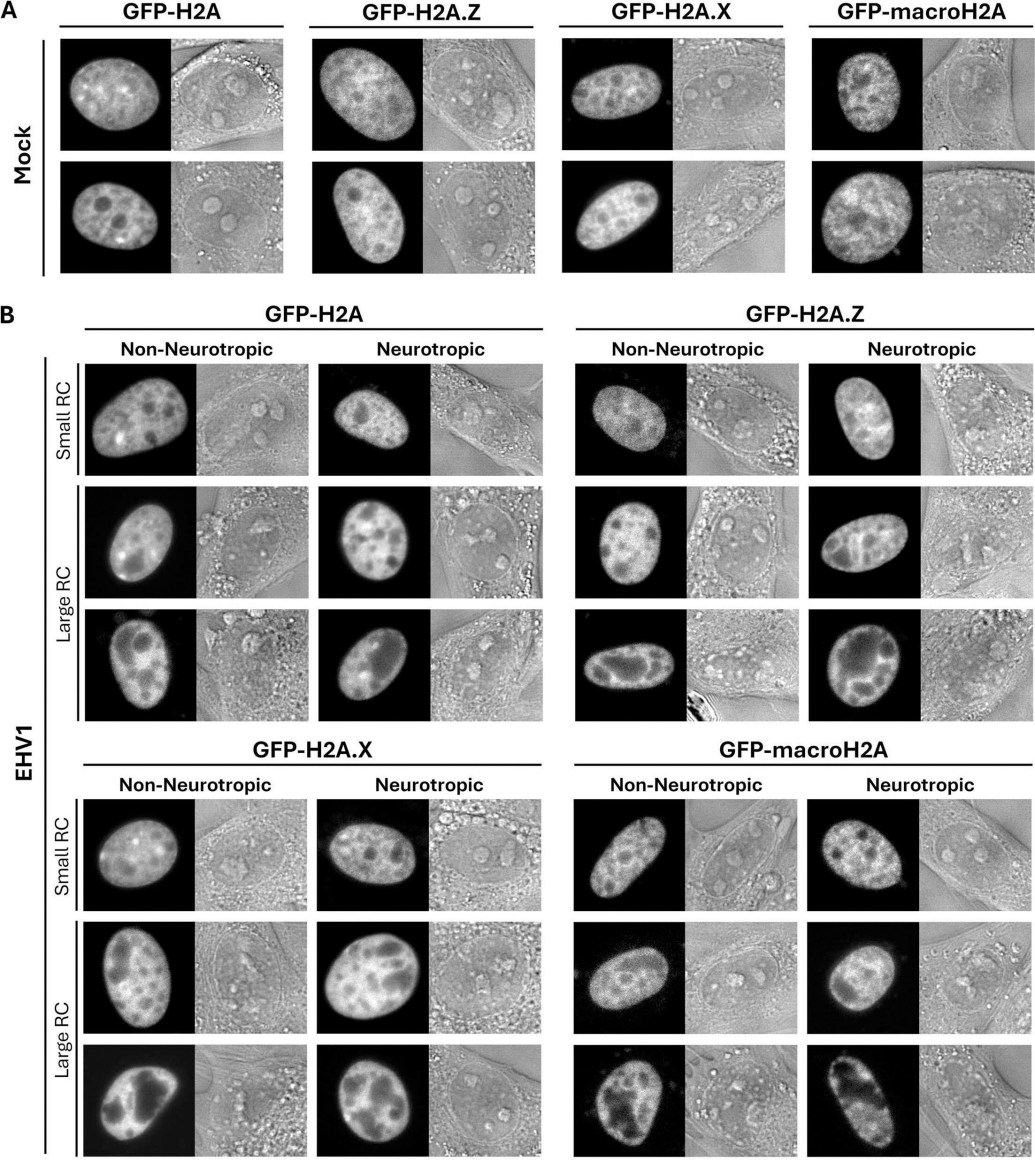
Canonical H2A and variant H2A.Z, H2A.X, and macroH2A are largely depleted from RCs. Digital fluorescent (left panels) and DIC (right panels) micrographs show the nucleus of EDerm cells expressing GFP-H2A, -H2A.Z, -H2A.X, or -macroH2A. Cells were mock-infected (**A**) or infected with 10 PFU/cell of non-neurotropic or neurotropic EHV1 (**B**) and live cells imaged between 5 and 6 hpi. As described in Fig. 2 and 7, nucleoli are visible as discrete domains in DIC images that correspond to regions variably depleted of GFP-histones in fluorescent images. EHV1 RCs are visible as regions variably depleted for GFP-histones in fluorescent images and are not visible in DIC images. Fluorescent images presented in grayscale for visibility.

To further interrogate whether EHV1 preferentially mobilizes particular histone variants, we next evaluated mobility of equine canonical H2A and variant H2A.Z, H2A.X, and macroH2A during EHV1 infection (the uniquely mobile H2A.B variant is presented and discussed below). Transiently expressed GFP-H2A, -H2A.Z, -H2A.X, and -macroH2A had discrete granular nuclear localization consistent with their assembly in chromatin (Fig. 9A). Regions of varying fluorescence intensity marked chromatin regions relatively enriched or depleted for each histone (Fig. 9A). Variant and canonical H2A were typically largely depleted from nucleoli, although H2A and H2A.Z were often less depleted than H2A.X or macroH2A (Fig. 9A; compare nucleolar regions visible in fluorescent and DIC images). Consistent with other evaluated core histones, H2A, H2A.Z, H2A.X, and macroH2A were also largely depleted from EHV1 RCs (Fig. 9B). RCs are identified as GFP-histone depleted regions in fluorescent images that do not correspond to visible domains in DIC images (for examples, see regions marked by white asterisks in Fig. 2B and 7B). Conversely, nucleoli in EHV1-infected cells are identified as GFP-histone depleted regions that correspond to visible domains in DIC images (for examples, see regions marked by arrows in Fig. 2B and 7B).

**Fig 10:**
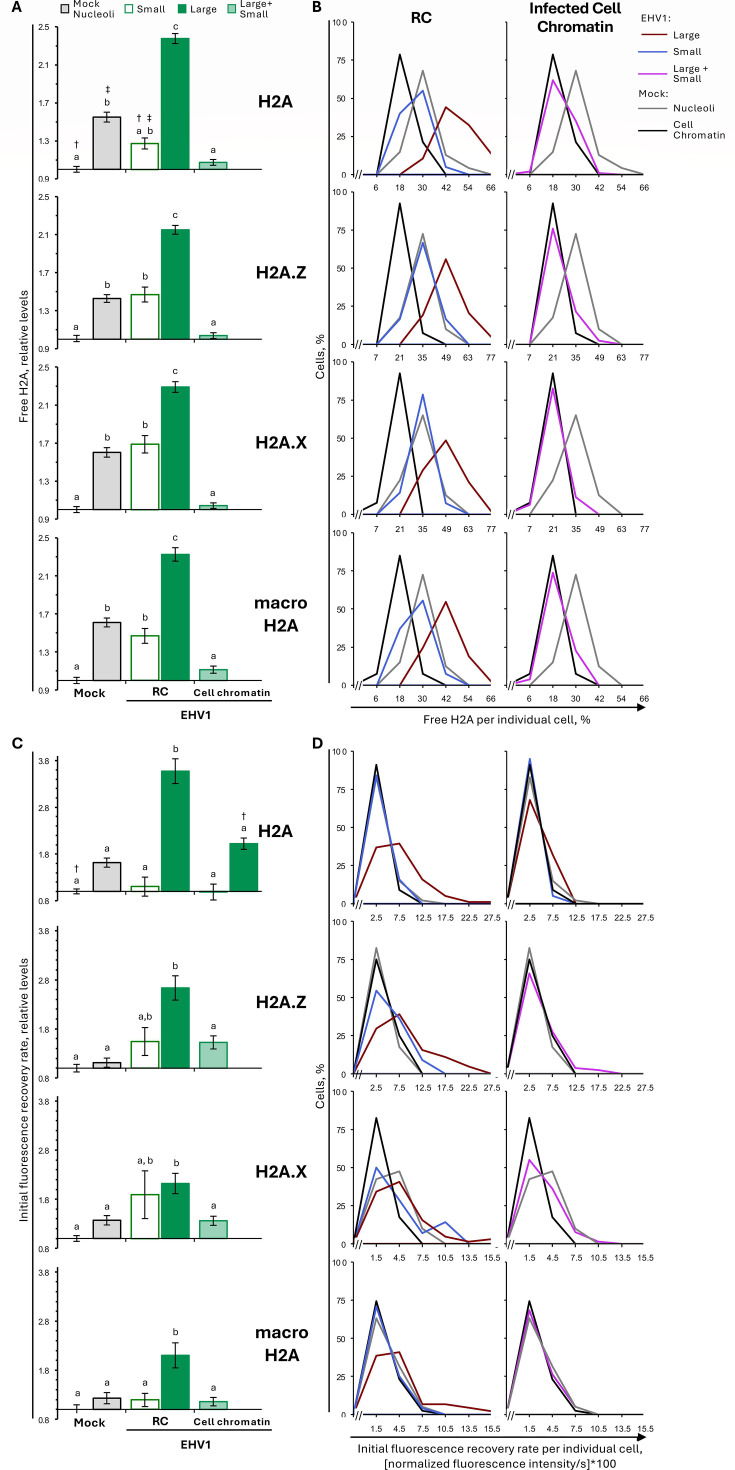
Canonical H2A and variant H2A.Z, H2A.X, and macroH2A are most mobile within “large” RCs. EDerm cells transfected with plasmids expressing GFP-H2A, -H2A.Z, -H2A.X, or -macroH2A were mock-infected or infected with 10 PFU/cell of non-neurotropic or neurotropic EHV1 at least 40 h after transfection. GFP-H2A, -H2A.Z, -H2A.X, or -macroH2A nuclear mobility was evaluated by FRAP between 5 and 6 hpi. Data for EHV1 infected cells were pooled for each histone and segregated by the absence (small) or presence (large) of clearly identifiable RCs (for examples, see Fig. 9B). (**A**) Bar graphs present the average GFP-H2A, -H2A.Z, -H2A.X, or -macroH2A normalized free pools expressed relative to their average normalized free pool in mock-infected cell chromatin (set at 1). (**B**) Frequency distribution plots show the percentage of free GFP-H2A, -H2A.Z, -H2A.X, or -macroH2A per individual cell. (**C**) Bar graphs present the average GFP-H2A, -H2A.Z, -H2A.X, or -macroH2A initial normalized fluorescence recovery expressed relative to their average initial normalized fluorescence recovery in mock-infected cell chromatin (set at 1). (**D**) Frequency distribution plots present the GFP-H2A, -H2A.Z, -H2A.X, or -macroH2A initial normalized fluorescence recovery per individual cell. (**B, D**) Data for mock-infected cells plotted in both graphs for comparison. Error bars, SEM. H2A n ≥ 47 cells per treatment from five independent experiments; H2A.Z, H2A.X, macroH2A n ≥ 40 cells per treatment from four independent experiments. Different letters denote *P* < 0.01; matching symbols denote *P* < 0.05. Statistical significance evaluated by ANOVA with post-hoc Tukey Kramer pair-wise analysis.

H2A variants are the most structurally and functionally diverse to regulate nucleosome stability and DNA accessibility. FRAP analysis revealed that, as expected, canonical and variant GFP-H2A fusion proteins had distinct mobilities within cell chromatin that correspond to their effects on nucleosome stability (Fig. S4) (27, 29, 30, 33, 35, 57, 64, 84). GFP-H2A.Z was the most mobile variant with a significantly larger free pool and increased fast chromatin exchange relative to canonical H2A (116% ± 3% and 144% ± 12%, respectively, *P* < 0.01; Fig. S4; Table S2). Conversely, GFP-macroH2A was the least mobile variant. Its free pool or fast chromatin exchange, however, was not significantly different from those of canonical H2A (91% ± 3% or 95% ± 10%, respectively; Fig. S4; Table S2). H2A, H2A.Z, H2A.X, and macroH2A were all more mobile within nucleoli and, surprisingly, were similarly mobile within this domain (Fig. 10; Fig. S4; Table S2). Only macroH2A fast chromatin exchange was relatively slower than that of H2A within nucleoli (76% ± 7%, *P* < 0.05; Fig. S4; Table S2).

EHV1 mobilized H2A, H2A.Z, H2A.X, and macroH2A within RCs (Fig. 10; Fig. S4). In “small” RCs, H2A, H2A.Z, H2A.X, or macroH2A free pools increased to 127% ± 6%, 147% ± 8%, 169% ± 9%, or 147% ± 8%, respectively (*P* < 0.01, except H2A *P* < 0.05; Fig. 10A; Table 3). This equated to a large increase in the free pools within “small” RCs in more than 50% of cells. H2A, H2A.Z, H2A.X, and macroH2A were further mobilized within “large” RCs, where their free pools increased to 238% ± 5%, 215% ± 5%, 229% ± 6%, or 233% ± 7%, respectively (*P* < 0.01; Fig. 10A; Table 3). This corresponded to a large increase in free pools within “large” RCs throughout the entire infected cell population (Fig. 10A and B; Table 3). Moreover, greater than 70% of cells had canonical or variant H2A free pools within “large” RCs greater than 1 SD above their average levels in mock-infected nucleoli. Consistently, H2A, H2A.Z, H2A.X, and macroH2A free pools within “large” RCs were significantly larger than those in mock-infected nucleoli. The high levels of free H2A, H2A.Z, H2A.X, or macroH2A were not due to the GFP-histone expression levels (Fig. S5A).

In “small” RCs, canonical or variant H2A free pools increased without substantial change to their fast chromatin exchange (Fig. 10C and D; Table 4). Only H2A.X fast chromatin exchange increased within “small” RCs and only in a minor cell subpopulation, shown by the bimodal frequency distribution of initial fluorescence recovery per individual cell (Fig. 10D; Table 4). Mobilization of canonical or variant H2A within “large” RCs, however, did increase their fast chromatin exchange. H2A, H2A.Z, H2A.X, or macroH2A fast chromatin exchange increased to 357% ± 27%, 263% ± 25%, 212% ± 21%, or 210% ± 26%, respectively, within “large” RCs (*P* < 0.01; Fig. 10C and D; Table 4).

Variant and canonical H2A were not significantly mobilized within the infected-cell chromatin. Free H2A, H2A.Z, H2A.X, or macroH2A pools were similar in EHV1- and mock-infected cell chromatin (Fig. 10A and B; Table 3). Their fast chromatin exchange was also largely unaltered. Only H2A fast chromatin exchange increased to 202% ± 12%, and only in cell chromatin surrounding “large” RCs (*P* < 0.05; Fig. 10C and D; Table 4).

In summary, EHV1 mobilized H2A, H2A.Z, H2A.X, and macroH2A. These histones were primarily mobilized in domains enriched for EHV1 chromatin (RCs). Mobilization of H2A, H2A.Z, H2A.X, and macroH2A within “large” RCs increased their free pools and fast chromatin exchange, whereas mobilization in “small” RCs primarily increased their free pools. Within “large” RCs, variant and canonical H2A were more mobile than in nucleolar chromatin, further supporting that EHV1 chromatin is highly dynamic. EHV1 largely did not alter variant or canonical H2A mobility within infected cell chromatin. Only canonical H2A was mobilized in chromatin surrounding “large” RCs. In this case, mobilization increased its fast chromatin exchange without affecting its free pool.

### EHV1 differentially mobilizes variant and canonical H2A

We next examined the mobility of variant relative to canonical H2A, as it was possible that EHV1 differentially mobilized particular H2A variants. In “small” EHV1 RCs, the intrinsic relationships between variant and canonical H2A mobility were poorly conserved. H2A.X was mobilized to the greatest degree such that it resembled more H2A.Z than H2A (Fig. 10; Fig. S4; Table S2). The H2A.X free pool increased to 133% ± 7% (*P* < 0.01) and its fast chromatin exchange rate tended to increase (to 136% ± 29%; *P* = ns) relative to H2A (Fig. S4; Table S2). H2A.Z was also further mobilized relative to H2A within “small” RCs. H2A.Z fast chromatin exchange increased from 144% ± 12% that of H2A within mock-infected cell chromatin to 172% ± 26% that of H2A within “small” RCs (Fig. S4; Table S2). However, despite increased fast chromatin exchange, H2A.Z free pools remained proportionately similar to those of H2A (131% ± 8% or 116% ± 3% that of H2A within “small” RCs or mock-infected cell chromatin, respectively; Fig. S4; Table S2).

Further mobilization of variant and canonical H2A in “large” RCs resulted in relatively similar free pools of H2A, H2A.X, and H2A.Z (Fig. S4; Table S2). However, H2A fast chromatin exchange increased by a greater magnitude than that of H2A.Z or H2A.X. Thus, H2A.Z fast chromatin exchange was similar to (117% ± 9%; *P* = ns), whereas that of H2A.X was again significantly slower than (66% ± 6%; *P* < 0.01) that of H2A within “large” RCs (Fig. S4; Table S2). Although macroH2A was further mobilized within “large” RCs, it remained the least mobile variant within them. Its free pool or fast chromatin exchange rate were 89% ± 2% or 64% ± 8% those of H2A, respectively (*P* < 0.01; Fig. S4; Table S2).

EHV1 infection had little effect on the intrinsic relationships between variant and canonical H2A mobility within infected-cell chromatin. However, as in “large” RCs, H2A.X and macroH2A fast chromatin exchange further decreased relative to H2A (102% ± 7% to 84% ± 5% or 95% ± 10% to 69% ± 5%, *P* < 0.05 or <0.01, respectively), consistent with H2A mobilization within cell chromatin surrounding “large” RCs (Fig. 10C; Fig. S4; Table S2). Nonetheless, the H2A, H2A.Z, H2A.X, and macroH2A free pools were proportionately maintained within infected-cell chromatin (Fig. S4).

In summary, EHV1 differentially mobilized canonical H2A and variant H2A.Z, H2A.X, and macroH2A within domains enriched for viral chromatin. H2A.X and H2A.Z were initially mobilized by a relatively greater degree within “small” RCs, suggesting that they are preferentially mobilized or more susceptible to mobilization factors in abundance at earlier infection stages. Mobilization of H2A.X and H2A.Z in “small” RCs, however, was distinct in that the H2A.X free pool was most increased, whereas H2A.Z fast chromatin exchange was most increased. The further mobilization of H2A, H2A.X, and H2A.Z at later infection stages resulted in relatively similar free pool levels within “large” RCs. However, despite similar free pools, H2A.X fast chromatin exchange was relatively decreased. MacroH2A was the least mobile variant in “large” RCs, with a relatively smaller free pool and slower fast chromatin exchange.

### Equine H2A.B is uniquely mobilized by EHV1

**Fig 11:**
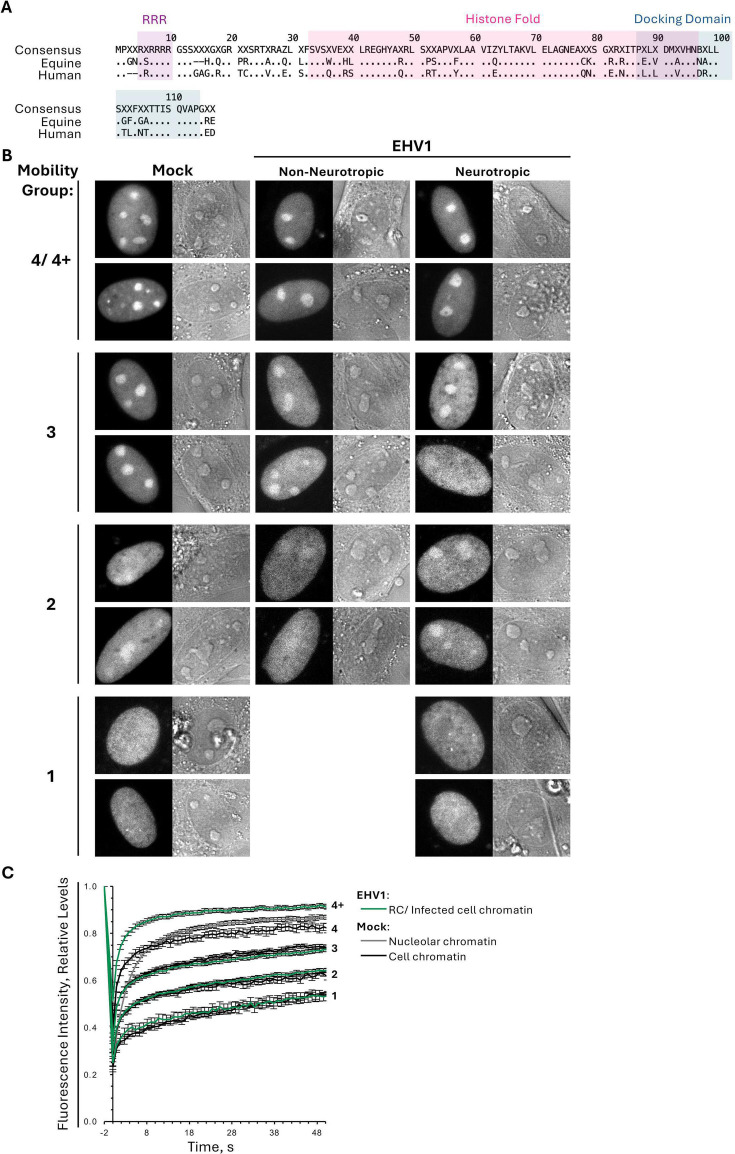
Variant H2A.B is neither depleted nor enriched in RCs. EDerm cells transfected with plasmids encoding GFP-H2A.B were mock-infected or infected with 10 PFU/cell of non-neurotropic or neurotropic EHV1 at least 40 h after transfection. Live cells were imaged and GFP-H2A.B mobility evaluated by FRAP between 5 and 6 hpi. FRAP data from EHV1-infected cells or the cell chromatin of mock-infected cells were combined and segregated into mobility groups (Fig. S6). (**A**) Amino acid sequences of equine and human H2A.B (XP_023489462.1 or NP_001017991, respectively). Sequences pairwise aligned using MUSCLE Alignment in Geneious Prime 2025.1.2. Identical residues indicated by a dot. Highlighted residues mark the arginine repeat (purple), histone fold (pink), and docking domain (blue) as denoted for human H2A.B on HistoneDB 2.0 (87). (**B**) Digital fluorescent (left panels) and DIC (right panels) micrographs show the nucleus of cells expressing GFP-H2A.B. Note that nucleoli are visible as discrete domains in DIC images that correspond to regions variably enriched for GFP-H2A.B in fluorescent images. RCs are not visible in fluorescent or DIC images. Fluorescent images presented in grayscale for visibility. (**C**) Line graph presents FRAP of GFP-H2A.B. Error bars, SEM; n ≥ 38 cells per treatment from four independent experiments.

**Fig 12:**
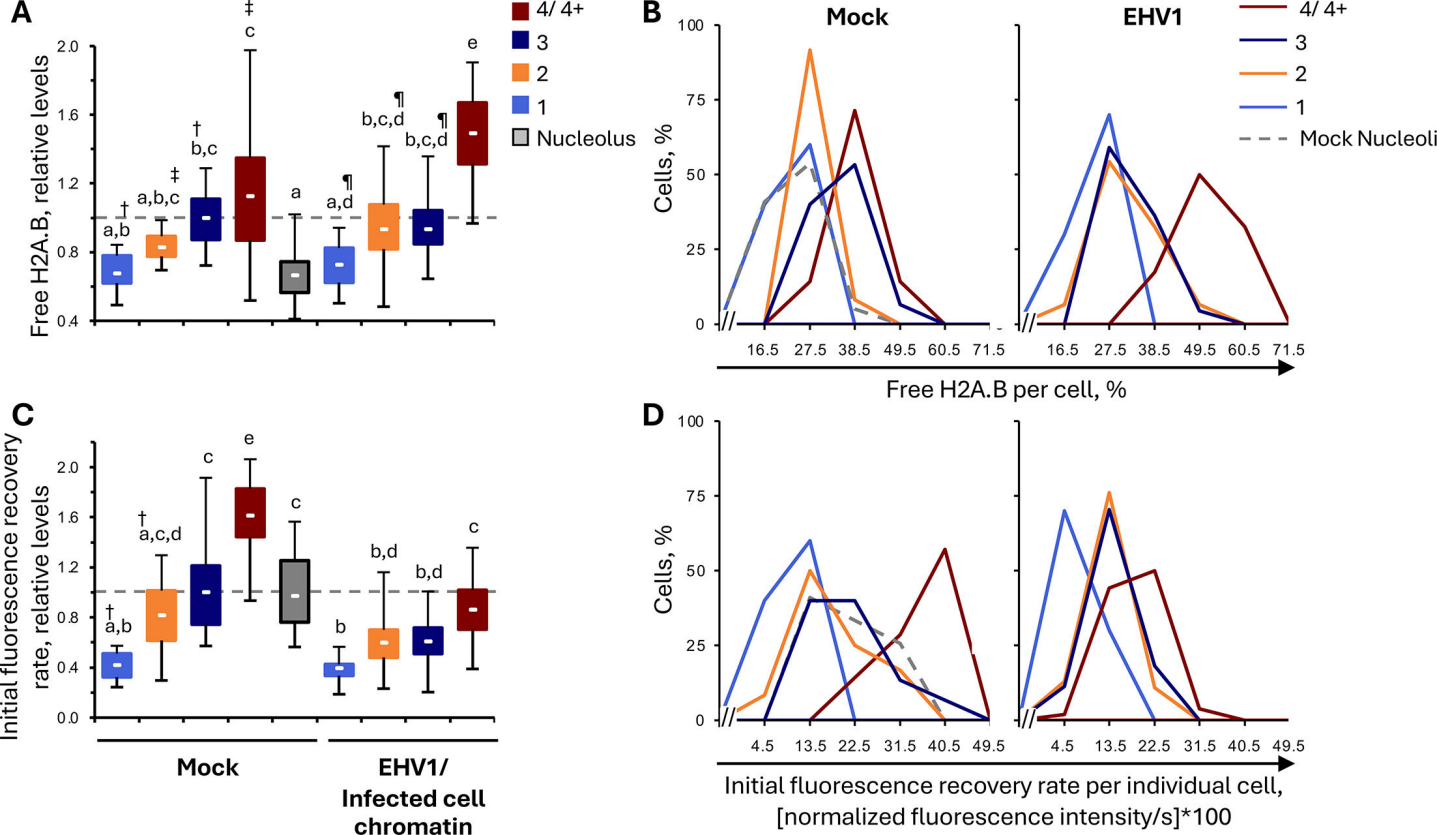
H2A.B is mobilized in a subpopulation of EHV1-infected cells. EDerm cells transfected with plasmids encoding GFP-H2A.B were mock-infected or infected with 10 PFU/cell of non-neurotropic or neurotropic EHV1 at least 40 h after transfection. GFP-H2A.B nuclear mobility was evaluated by FRAP between 5 and 6 hpi. FRAP data were pooled for EHV1-infected cells or mock-infected cell chromatin and segregated by mobility group. (**A**) Box and whisker plot shows the average GFP-H2A.B normalized free pool per mobility group expressed relative to the average normalized free pool in mock-infected cell chromatin of mobility group 3 (set at 1). (**B**) Frequency distribution plots show the percentage of free GFP-H2A.B per individual cell segregated by mobility group. (**C**) Box and whisker plot shows the average GFP-H2A.B initial normalized fluorescence recovery per mobility group expressed relative to the average initial normalized fluorescence recovery in mock-infected cell chromatin of mobility group 3 (set at 1). (**D**) Frequency distribution plots present the GFP-H2A.B initial normalized fluorescence recovery per individual cell segregated by mobility group. (**A, C**) Boxes encompass 25th to 75th percentiles; line, average; top whisker, highest value; bottom whisker, lowest value. n ≥ 38 cells per treatment from four independent experiments. Different letters denote *P* < 0.01; matching symbols denote *P* < 0.05. Statistical significance evaluated by ANOVA with post-hoc Tukey Kramer pair-wise analysis.

H2A.B is the most unique and dynamic somatic H2A variant and is poorly conserved among species (86). Equine and human H2A.B share only 70% sequence identity (Fig. 11A). Human H2A.B is uniquely mobilized by HSV1 (57). It is relatively demobilized along with accumulation in HSV1 RCs and enrichment in HSV1 chromatin (57). We therefore evaluated whether EHV1 would dysregulate equine H2A.B mobility in a similar manner. Equine H2A.B had distinct nuclear localization patterns that ranged from largely homogenous throughout the nucleus to pronounced enrichment within nucleoli (Fig. 11B). The variable localization of equine H2A.B resembled cell cycle-related localization of human H2A.B and likewise may relate to cell cycle stage (44). Post hoc analysis of the FRAP of each individual cell revealed that H2A.B mobility within non-nucleolar chromatin naturally segregated into mobility groups that correspond to localization patterns (Fig. 11B and C). We arbitrarily numbered these mobility groups from the slowest (1), which corresponded to relatively homogenous H2A.B distribution, to the fastest (4), which corresponded to pronounced nucleolar H2A.B enrichment (Fig. 11B and C). Most cells were within mobility groups 3 (38%) and 2 (31%), with smaller populations within the most extreme mobility groups 1 (13%) and 4 (18%) (Fig. 11; Fig. S6; Table S3). The slowest mobility group one had the smallest H2A.B free pool and slowest fast chromatin exchange, whereas the fastest mobility group four had the largest and fastest, respectively (Fig. 12; Tables 3 and 4). The variable mobility of H2A.B within non-nucleolar chromatin did not relate to GFP-H2A.B expression levels (Fig. S5B).

H2A.B was similarly mobile within nucleoli of all mock-infected cells (Fig. 11C; Fig. S6C). The similar mobility of H2A.B within nucleoli, regardless of its mobility within the cell chromatin, further supports the independence of measured mobility and GFP-H2A.B expression levels. Additionally, these data show that H2A.B mobility within nucleoli is independent of nucleoli number or their relative H2A.B enrichment. The H2A.B free pool within nucleoli was similar to that within the surrounding cell chromatin for mobility groups 1 and 2 (67% ± 2%, 67% ± 6%, and 83% ± 3%, respectively, of the H2A.B free pool within mobility group 3; Fig. 12; Table 3). H2A.B fast chromatin exchange within nucleoli, however, resembled more that of mobility group 3, which had a significantly larger free pool (Fig. 12; Tables 3 and 4).

H2A.B is the most dynamic H2A variant (44, 48, 60, 61, 63, 84). Consistently, within non-nucleolar cell chromatin, all H2A.B mobility groups had larger free pools (with the exception of mobility group 1) and increased fast chromatin exchange relative to canonical H2A (Fig. S4; Table S2). Within nucleoli, however, the H2A.B free pool was significantly smaller (74% ± 2%) and its fast chromatin exchange significantly faster (567% ± 30%) relative to canonical H2A (*P* < 0.01, Fig. S4; Table S2).

EHV1 infection surprisingly did not alter H2A.B nuclear localization. H2A.B still had varying localization patterns ranging from largely homogenous throughout the nucleus to pronounced nucleolar enrichment (Fig. 11B). Visible H2A.B depletion from, or enrichment in, EHV1 RCs was never observed. Nuclear domains likely to be enriched in viral- or cell-chromatin were therefore indistinguishable, and measured H2A.B mobility represents its net mobility due to exchange with cell- and EHV1-chromatin. H2A.B also consistently segregated into mobility groups within infected cells (Fig. 11C). The slowest H2A.B mobility groups within infected cells (1–3) were similar to those within mock-infected cell chromatin (Fig. 11C and 12; Fig. S6). Only the fastest mobility group within EHV1-infected cells, designated as 4+, was mobilized relative to mock-infected cells. This most mobile H2A.B was evident regardless of whether cells were infected with non-neurotropic or neurotropic EHV1, although the relative population of cells within this, or the slower mobility groups, varied (Fig. S6; Table S3). Most non-neurotropic EHV1-infected cells segregated into mobility group 4+, with no cells in the slowest mobility group 1 (Fig. S6; Table S3). Meanwhile, most neurotropic EHV1-infected cells segregated into mobility groups 2 and 3 (Fig. S6; Table S3). H2A.B mobility may thus relate to infection progression as non-neurotropic EHV1 tended to have slightly faster replication kinetics in EDerm cells (data not shown). Alternatively, other factors, such as specific virus-host protein-protein interactions, may contribute to regulating H2A.B mobility during EHV1 infection.

H2A.B mobility group 4+ was the most mobile. Fluorescence within these cells recovered faster than within any other mobility group of mock- or EHV1-infected cells or within mock-infected nucleoli (Fig. 11C). H2A.B mobility group 4+ had the largest free pool (149% ± 3%) and the greatest fast chromatin exchange (86% ± 3%) in EHV1-infected cells (expressed relative to H2A.B mobility group 3 in mock-infected cell chromatin; Fig. 12; Tables 3 and 4). The H2A.B free pool within mobility group 4+ was also significantly increased relative to that within mock-infected mobility group 4 (149% ± 3% vs 113% ± 4%), although its fast chromatin exchange rate was significantly slower (86% ± 3% vs 161% ± 15%, *P* < 0.01; Fig. 12; Tables 3 and 4).

The inability to distinguish EHV1 RCs in H2A.B expressing cells precluded comparative analysis of H2A.B and H2A mobilization within domains enriched in EHV1 or infected-cell chromatin. As a surrogate analysis, H2A.B mobility was expressed relative to H2A mobility within infected-cell chromatin or “large” RCs. The intrinsic property of H2A.B as the most dynamic H2A variant was maintained relative to H2A within infected-cell chromatin (Fig. S4; Table S2). All H2A.B mobility groups had larger free pools (with the exception of mobility group 1) and increased fast chromatin exchange relative to H2A within infected-cell chromatin (Fig. S4; Table S2). With respect to H2A mobility within “large” RCs, however, only H2A.B mobility group 4+ had a larger free pool (108% ± 2%, *P* < 0.05) and increased fast chromatin exchange (257% ± 8%, *P* < 0.01) relative to H2A (Fig. S4; Table S2). Mobility groups 1–3 had significantly smaller free pools and, with the exception of mobility group 1, significantly increased fast chromatin exchange relative to H2A within “large” RCs (Fig. S4; Table S2). The relative mobilities of H2A.B mobility groups 1–3 and H2A within “large” RCs were similar to their relative mobilities within mock-infected nucleolar chromatin.

These data highlight the unique mobility of equine H2A.B. EHV1 did not notably alter H2A.B nuclear localization and mobilized it only within a subpopulation of infected cells (4+). Within this subpopulation, H2A.B free pools increased despite a relative decrease in fast chromatin exchange.

### Equine linker histone H1.2 is most mobile within RCs

**Fig 13:**
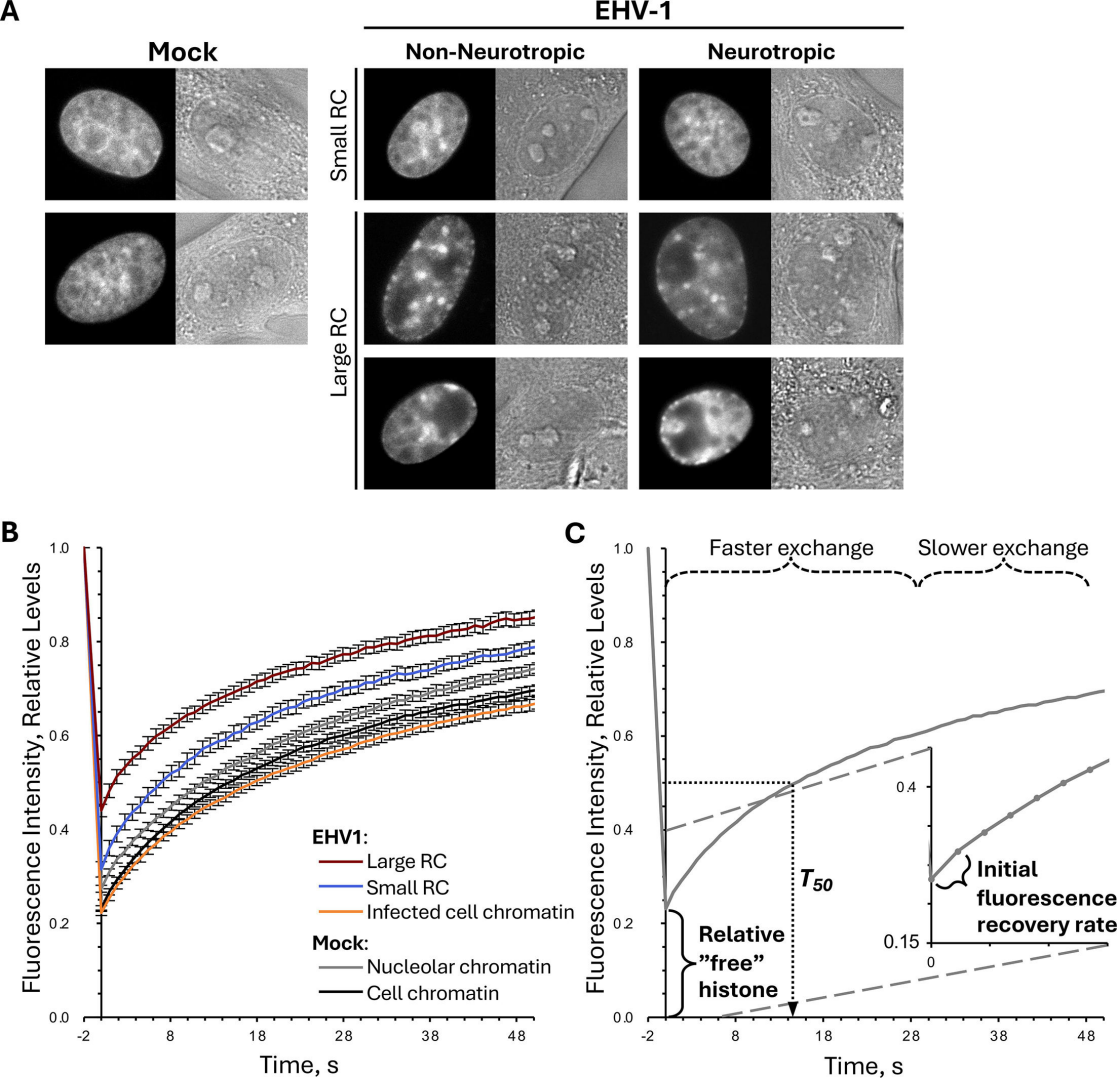
EHV1 mobilizes linker histone H1.2. EDerm cells transfected with plasmids encoding GFP-H1.2 were mock-infected or infected with 10 PFU/cell of non-neurotropic or neurotropic EHV1 at least 40 h after transfection. Live cells were imaged and GFP-H1.2 mobility evaluated by FRAP between 5 and 6 hpi. FRAP data for each EHV1-infected cell were combined and segregated by the presence (large) or absence (small) of clearly identifiable RCs, examples of which are shown in (**A**). (**A**) Digital fluorescent (left panels) and DIC (right panels) micrographs show the nucleus of cells expressing GFP-H1.2. Fluorescent images presented in grayscale for visibility. (**B**) Line graph presents FRAP of GFP-H1.2. (**C**) Line graph of a representative GFP-H1.2 FRAP in mock-infected cell chromatin (an area marked by solid yellow circle in Fig. 3A). The surrogate measures for H1.2 free pool levels and fast chromatin exchange are calculated as for core histones (described in Fig. 3B). The *T_50_* is a summary measure that primarily reflects weak chromatin interactions. It is the time required to recover 50% normalized fluorescence in the photobleached region. Error bars, SEM; n ≥ 38 cells per treatment from four independent experiments.

Linker histones are most dynamic and undergo rapid chromatin exchange (54, 59–62). We evaluated equine H1.2 mobility during EHV1 infection as a representative linker histone, given that it is abundantly expressed throughout the cell cycle, assembles in euchromatin, and is the H1 variant most mobilized by HSV1 (Fig. S1) (54, 62, 88). Equine GFP-H1.2 had discrete granular nuclear distribution with relative nucleolar depletion (Fig. 13A). However, its depletion from nucleoli tended to be less pronounced than for most core histones (Fig. 13A). FRAP analysis revealed that, as expected, H1.2 was more mobile than core histones and also more mobile within nucleoli than within surrounding cell chromatin (Fig. 13B Mock).

EHV1 altered H1.2 localization such that it was relatively depleted from RCs and accumulated in discrete puncta within infected-cell chromatin (Fig. 13A). H1.2 redistribution was reflected in its mobility. H1.2 was mobilized within RCs, where it was more mobile in “large” than in “small” ones (Fig. 13B). Conversely, H1.2 mobility decreased in infected-cell chromatin such that fluorescence recovered slower than within mock-infected cell chromatin (Fig. 13B). The apparent H1.2 demobilization in infected-cell chromatin is consistent with its accumulation (or immobilization) in discrete puncta and the robust cellular chromatin condensation observed by Hoechst staining in fixed cells (Fig. 1 and 13A).

**Fig 14:**
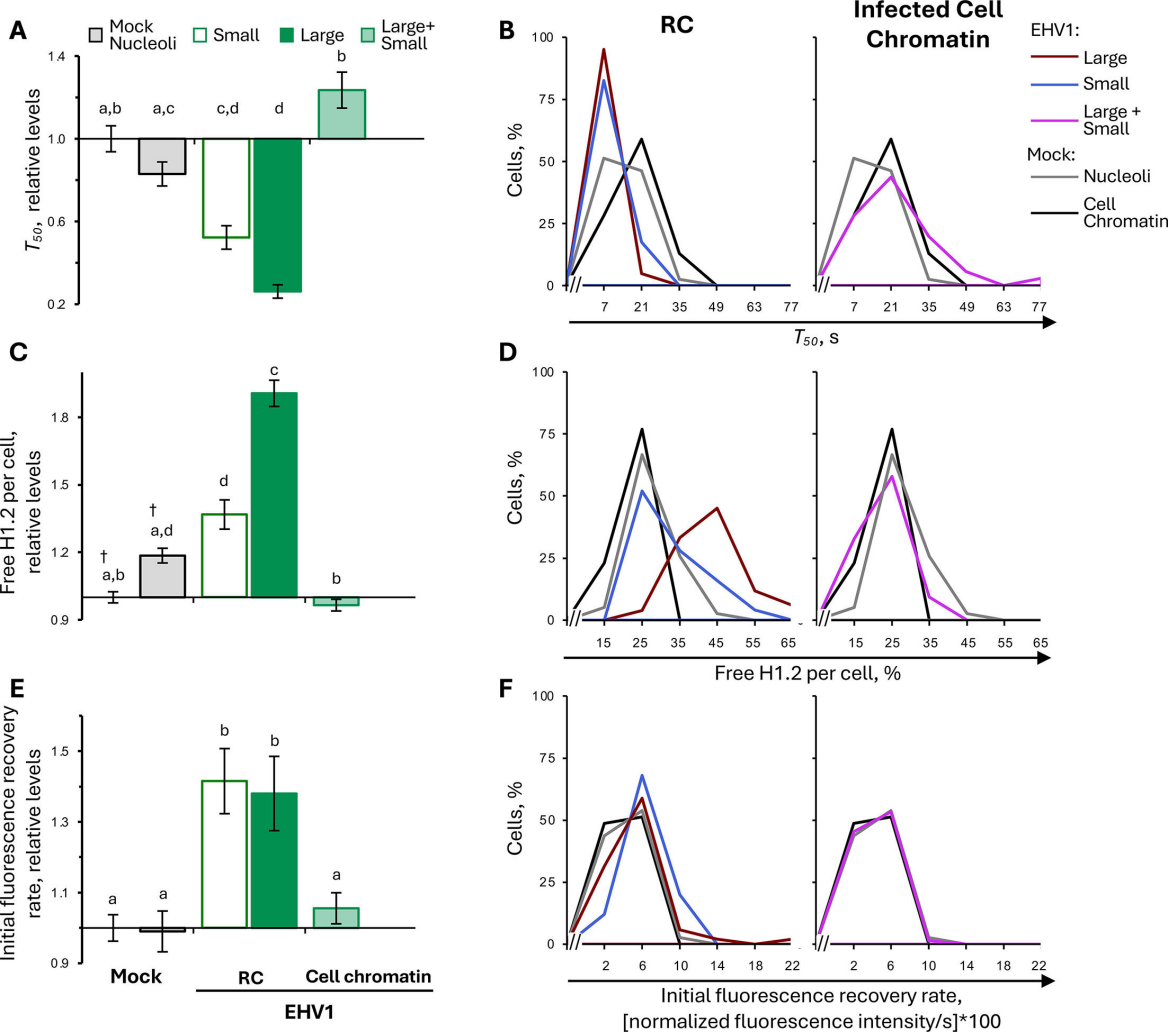
H1.2 is most mobile in RCs. EDerm cells transfected with plasmids encoding GFP-H1.2 were mock-infected or infected with 10 PFU/cell of non-neurotropic or neurotropic EHV1 at least 40 h after transfection. GFP-H1.2 nuclear mobility was evaluated by FRAP between 5 and 6 hpi. FRAP data for each EHV1-infected cell were pooled and segregated by the absence (small) or presence (large) of clearly identifiable RCs (for examples see Fig. 13A). (**A**) Bar graph presents the average GFP-H1.2 *T_50_* expressed relative to its average *T_50_* in mock-infected cell chromatin (set at 1). (**B**) Frequency distribution plots present the GFP-H1.2 *T_50_* per individual cell. (**C**) Bar graph presents the average GFP-H1.2 normalized free pool expressed relative to its average normalized free pool in mock-infected cell chromatin (set at 1). (**D**) Frequency distribution plots show the percentage of free GFP- H1.2 per individual cell. (**E**) Bar graph presents the average GFP-H1.2 initial normalized fluorescence recovery expressed relative to its average initial normalized fluorescence recovery in mock-infected cell chromatin (set at 1). (**F**) Frequency distribution plots present the GFP-H1.2 initial normalized fluorescence recovery per individual cell. (**B, D, F**) Data for mock-infected cells plotted in both graphs for comparison. Error bars, SEM. n ≥ 38 cells per treatment from four independent experiments. Different letters denote *P* < 0.01; matching symbols denote *P* < 0.05. Statistical significance evaluated by ANOVA with post-hoc Tukey Kramer pair-wise analysis.

As an overall measure of H1.2 mobility, we calculated its *T_50_*, the time to recover 50% normalized fluorescence intensity within a photobleached region (Fig. 13C). *T_50_* and mobility inversely relate such that a smaller *T_50_* reflects increased mobility. *T_50_* is most influenced by weaker chromatin interactions and primarily reflects mobility of the most dynamic histones. H1.2 mobilization in “small” or “large” RCs decreased its *T_50_* to 52% ± 6% or 26% ± 3%, respectively, of that in mock-infected cell chromatin (*P* < 0.01; Fig. 14A and B). H1.2 was mobilized within RCs throughout the infected cell population with a large degree of mobilization in 61% or 93% of “small” or “large” RCs, respectively (Fig. 14B). H1.2 mobilization within RCs caused a net increase in its free pools, to 137% ± 6% or 191% ± 6%, and increased its fast chromatin exchange, to 142% ± 9% or 138% ± 11%, within “small” or “large” RCs, respectively (*P* < 0.01; Fig. 14C through F; Tables 3 and 4). Interestingly, H1.2 fast chromatin exchange in “small” or “large” RCs was similar despite a significantly larger H1.2 free pool within the “large” ones (Fig. 14C and E).

In contrast to mobilization within RCs, H1.2 mobility was decreased within infected-cell chromatin (Fig. 13B). Reduced H1.2 mobility tended to increase its *T_50_* to 124% ± 9%, although significance was not achieved (Fig. 14A and B). Regardless, 31% of cells had a *T_50_* greater than 1 SD above the average *T_50_* in mock-infected cell chromatin, which is almost double the expected population were H1.2 not immobilized within it (Fig. 14B). Although overall H1.2 mobility decreased in EHV1-infected cell chromatin, its free pool and fast chromatin exchange were similar to mock-infected cell chromatin (97% ± 3% or 106% ± 4%, respectively; Fig. 14C through F; Tables 3 and 4). These data suggest that decreased H1.2 mobility within infected-cell chromatin reflects further stabilization of stable, rather than weak, H1.2 chromatin interactions.

Together, these data show that EHV1 mobilized H1.2 within domains enriched for EHV1 chromatin (RCs). H1.2 mobilization was apparently independent of infection stage, although at later stages H1.2 free pools were increased. EHV1 decreased H1.2 mobility within infected-cell chromatin. De-mobilization of H1.2 did not affect its free pool or fast chromatin exchange, suggesting that EHV1 may further stabilize stable H1.2 interactions in infected-cell chromatin.

## DISCUSSION

We show that EHV1 mobilized equine canonical and variant core histones and linker histone H1.2 during infection of equine cells. With the exception of H2A.B, all histones were most mobile within RCs where EHV1 chromatin is enriched. However, histones were not equally mobilized within RCs, nor were they consistently mobilized over the course of infection. Canonical and variant H2A (with exception of H2A.B), H2B, and H4 mobilization at earlier infection stages primarily increased their free pools, whereas their further mobilization at later stages increased their free pools and fast chromatin exchange (Fig. 6 and 10). Conversely, canonical H3.1 and variant H3.3 were not significantly mobilized at earlier infection stages but were at later stages. In this case, mobilization increased their free pools and differentially altered their fast chromatin exchange (Fig. 8). Meanwhile, linker histone H1.2 was largely fully mobilized at earlier infection stages (Fig. 14). The apparent further mobilization at later stages resulted from higher free pools in “large” RCs. The distinct mobilities of individual histone types are consistent with a model in which different mechanisms specifically mobilize individual histones. Further in support of this model was the preferential mobilization of individual variant or canonical histones. H2A.X and H2A.Z were preferentially mobilized at earlier infection stages. However, they were also distinctly mobilized in that H2A.X free pools relatively increased while H2A.Z fast chromatin exchange relatively increased (Fig. 10; Fig. S4). In “large” RCs, H2A and H3.3 were preferentially mobilized, which resulted in relatively increased fast chromatin exchange and, in the case of H3.3, an increased free pool (Fig. 8 and 10; Fig. S3 and S4). Specific mobilization of any individual histone variant may promote its assembly in viral chromatin. Conversely, mobilization may reflect eviction from, or inhibition of assembly in, EHV1 chromatin. For example, in “large” RCs, H3.3 and H3.1 had relatively similar fast chromatin exchange; however, the H3.3 free pool was significantly larger. This mobility suggests that H3.3 is less likely assembled in, or more likely evicted from, EHV1 chromatin than H3.1 (Fig. 8; Fig. S3). Similarly, mobility of H1.2 in “large” RCs was consistent with increased eviction from, or restricted assembly in, EHV1 chromatin. Together, these data show that variant and canonical histone mobility is uniquely altered and their relative mobilities change as infection progresses. Importantly, preferential and different mobilization of individual histones is consistent with varied EHV1 chromatin composition. Thus, EHV1 chromatin may be enriched in specific histones at different infection stages, as is HSV1 chromatin (56, 57, 89).

As for EHV1, canonical (H2A, H2B, H3.1, and H4) and variant (H2A.X, macroH2A, and H3.3) core histones are also more dynamic in HSV1 RCs than in nucleoli (K.L. Conn and L.M. Schang, unpublished data). The high mobility of histones within RCs is consistent with highly unstable and hyperaccessible viral nucleosomes (57, 77–80, 90–99). The functional relationships between histone mobility and viral nucleosome stability, however, are yet to be tested. It is possible that chromatin composition contributes more to nucleosome stability than histone mobility. H2A.B assembles highly unstable nucleosomes and is enriched within HSV1 chromatin (47, 56, 57, 89). Assembly in HSV1 chromatin is accompanied by H2A.B relative demobilization and accumulation in RCs. H2A.B mobility and sub-nuclear localization were largely unaltered by EHV1 infection. It was only mobilized (rather than immobilized) within a subpopulation of infected cells (Fig. 11 and 12). Moreover, H2A.B (or any other histone) was not enriched within EHV1 RCs. Equine and human H2A.B are the most dissimilar histones evaluated herein, sharing only 70% a.a. identity (Fig. 11A). It therefore is not entirely surprising that they are distinctly mobilized during infection. Differences in histone mobilization by EHV1 and HSV1 suggest that their chromatins have unique properties or are uniquely regulated. Although EHV1 chromatin composition and biophysical properties remain unknown, identified differences in EHV1 histone mobilization provide promising avenues to functionally test relationships between histone mobility and viral nucleosome stability.

The similar appearance of “small” RCs and histone-depleted regions within mock-infected cells confounded analysis of histone mobility at earlier infection stages. It is possible that measured mobility within “small” RCs is slower than reality due to unintentional selection of non-RC regions. However, bimodal frequency distributions for histone mobility within individual “small” RCs were only evident for H2B and H2A.X fast chromatin exchange (Fig. 6E and 10D). All other analyses produced unimodal frequency distributions intermediate to histone mobility in cell chromatin and “large” RCs. These data are consistent with a mixed population of cells with histones mobilized to varying degrees within any single “small” RC. Such mobilization supports a model where increased levels of EHV1 transcription, DNA replication, or expression of viral protein(s) directly or indirectly enhance histone mobility. This model is consistent with HSV1 histone mobilization, for which this phenomenon was first described (54). Although all HSV1 transcription activators mobilize histones, the IE protein ICP4 mobilizes them to the greatest degree and is sufficient to do so (54–56, 76). The EHV1 ICP4 homolog, IE1, shares only 34% a.a. homology with ICP4, yet has some conservation of its transcription regulatory mechanisms (100–104). Whether ICP4 histone mobilization activities are also conserved in IE1 remains unknown.

EHV1 altered mobility of some histones (H1.2, H2A, H3.3, and H4) within cell chromatin surrounding RCs. Mobilization of H4 increased its free pool and fast chromatin exchange, whereas that of H2A increased its fast chromatin exchange and that of H3.3 increased its free pool (Fig. 6, 8A and 10C). Conversely, H1.2 was relatively immobilized within cell chromatin surrounding RCs (Fig. 13). The different mobilization of individual histone types within infected-cell chromatin further supports that specific mechanisms target and regulate histone mobility in EHV1-infected cells. Furthermore, the distinct mobility of histones within RCs or infected-cell chromatin highlights that histone assembly and turnover in either chromatin are differentially regulated and suggest that EHV1 and cellular chromatin have unique properties.

Prominent differences in histone mobility during infection with non-neurotropic or neurotropic EHV1 were not apparent. However, several trends suggest differences in the chromatin regulation of either strain. For example, H2A.Z and H3.1 fast chromatin exchange tended to be slower in “small” and faster in “large” RCs during neurotropic EHV1 infection. H3.3 and H4 fast chromatin exchange also tended to increase in cell chromatin surrounding “large” RCs during neurotropic EHV1 infection. These trends may reflect aspects of viral chromatin regulation not readily apparent by FRAP analyses. Knowledge of the mechanisms that mobilize histones will support more in-depth characterization of EHV1 chromatin and its regulation and may highlight pathotype-specific mechanisms.

Histone depletion within RCs is consistent with their specific mobilization in EHV1 chromatin. However, minor cell subpopulations had relatively less H3.1, H3.3, or macroH2A depletion within RCs or diffuse H3.1 or H3.3 localization (Fig. 7 and 9). These data, in combination with H2A.B mobility and localization (Fig. 11), highlight that histones can be equally enriched and equally mobile within EHV1 and cellular chromatin at least in some scenarios. It is interesting that in such scenarios, histone interactions with nucleolar chromatin remained distinct. Nucleoli remained relatively depleted for H3.1 or H3.3 in cells with diffuse localization and remained relatively H2A.B enriched in cells that had mobilized it (mobility group 4+, Fig. 7B and 11B).

Herein, we show that nucleoli were not morphologically disrupted during EHV1 infection (Fig. 2, 3, 7, 9, 11, and 13). Nucleolar maintenance may account for particular differences in histone mobilization by EHV1 or HSV1. For example, H2A.B may preferentially assemble in nucleolar chromatin and, consequently, be less available or less likely to stably assemble in EHV1 chromatin. Conversely, HSV1-mediated nucleolar disruption could displace H2A.B from nucleolar chromatin to promote its viral chromatin assembly (46, 105–115). Additionally, two nucleolar resident proteins that redistribute throughout HSV1-infected nuclei, nucleolin and NPM1, are histone chaperones that exchange H2A-H2B or H3-H4 dimers and promote chromatin decondensation to support histone mobilization (105, 106, 109, 114–121). Increased histone chaperone abundance in HSV1-infected cell chromatin could mobilize histones within it. Consistently, canonical (H2A, H2B, H3.1, and H4) and variant (H2A.X, macroH2A, and H3.3) core histones are mobilized by HSV1 to increase their free pools within infected-cell chromatin (K. L. Conn and L. M. Schang, unpublished data). In contrast, EHV1 largely did not mobilize histones within cell chromatin. H2A, H3.3, and H4 were only partially mobilized, whereas H1.2 was relatively immobilized (Fig. 6, 8A, and 10C). Limited mobilization of histones in cell chromatin, in conjunction with notable cellular chromatin condensation (observed by Hoechst stain), indicates that equine and human cell chromatin are also uniquely affected by alphaherpesvirus infection. Distinct nuclear changes during EHV1 infection of equine cells highlight avenues for comparative analyses of alphaherpesvirus pathogenesis and cellular responses to infection. Such analyses provide opportunities to identify novel processes, or emphasize less prominent ones, to increase our overall understanding of virus-host interactions and how they ultimately influence infection outcome in various species.

Herein, we provide a comprehensive and comparative analysis of equine histone mobility within nucleolar and non-nucleolar chromatin of mock-infected equine cells. Importantly, as most chromatin investigations use primate- or murine-derived cells, our analysis in equine-derived cells provides a unique perspective and opportunity to identify chromatin processes that may be novel or differently regulated in more evolutionarily distant mammalian species. Equine core histone mobility reported herein is largely as expected with respect to histone effects on nucleosome stability. Furthermore, equine histone mobilities were also consistent with our understanding of chromatin stability and histone mobility in that all histones were more mobile within nucleolar than non-nucleolar chromatin. It is interesting to note that H2A variants, aside from H2A.B, were similarly mobile within nucleolar chromatin despite distinct mobilities within non-nucleolar chromatin (Fig. S4; Table S2). Likewise, H3.1 and H3.3 free pools were similar in nucleoli (Fig. S3; Table S2). H2A.B, as expected, was the most mobile core histone (Fig. 11 and 12; Fig. S4; Table S2). Within nucleoli, H2A.B had remarkably enhanced fast chromatin exchange but a significantly smaller free pool relative to H2A (Fig. S4; Table S2). These data indicate that H2A.B turnover in nucleolar chromatin is exceptionally rapid; however, H2A.B is also more likely to assemble in nucleolar chromatin than H2A. Together, these data highlight the unique properties and regulation of nucleolar chromatin.

We identified two examples of notable histone mobility within equine chromatin. The first relates to H2A.B mobility. To our knowledge, this study provides the first example of variable H2A.B mobility within non-nucleolar chromatin and distinct H2A.B mobility within nucleolar versus non-nucleolar chromatin (Fig. 11 and 12; Fig. S6). Variable and multiple H2A.B mobilities may reflect unique features of equine H2A.B. Alternatively, studies evaluating other species’ H2A.B may not have identified this phenomenon due to measuring mobility across larger nuclear regions that contain differing amounts of nucleolar and non-nucleolar chromatin (44, 48, 57). Regardless, variable mobility of equine H2A.B in non-nucleolar chromatin is consistent with multiple mechanisms to regulate it. Given the correlation between H2A.B mobility and sub-nuclear localization patterns, regulatory mechanisms may also relate to cell-cycle stage (44). Human H2A.B is subject to regulatory PTMs, including methylation of residues in the N-terminal arginine repeat and phosphorylation (122). The equine H2A.B arginine repeat is truncated from 6 to 4 residues, and it contains an additional serine residue (Fig. 11A). Differential PTMs of equine H2A.B may relate to differential mobilization or sub-nuclear localization in equine chromatin and may contribute to differences in H2A.B mobility across species. In contrast to non-nucleolar chromatin, H2A.B mobility in nucleolar chromatin was remarkably consistent (Fig. 11C; Fig. S6). This suggests that H2A.B mobility in nucleolar chromatin is tightly regulated and largely independent of factors that differentially regulate it in non-nucleolar chromatin. The second example of notable histone mobility relates to H3.1. To our knowledge, this is the first study to comparatively evaluate H3.1 and H3.3 mobility by FRAP. Comparative analysis revealed a population of H3.1 that was more dynamic than H3.3 within equine chromatin. A previous FRAP study that measured GFP-H3.1 or -H3.3 mobility also shows a more dynamic H3.1 population, although H3.3 and H3.1 were not compared directly (56). The reported GFP-H3.1 or -H3.3 free pools in Vero (African green monkey) cells were approximately 25% and <20%, respectively, which is comparable with the 25% and 21%, respectively, reported herein (Table 3) (56). Thus, our identification of a more mobile H3.1 population likely relates to the analysis technique rather than equine-specific chromatin regulation. FRAP is a sensitive technique that directly measures histone mobility in living cells. Other techniques that indirectly measure histone mobility typically use labeled histones to evaluate chromatin enrichment (or depletion) over time. Such approaches would not encounter most dynamic histones, as they are less efficiently fixed and commonly lost during isolation and preparation of nuclei.

In conclusion, we show that equine histones are highly mobile within EHV1 RCs. Such mobilization is consistent with the assembly of highly dynamic or unstable EHV1 nucleosomes. We propose that EHV1 histone mobilization represents a robust approach to counteract or prevent viral chromatin silencing. Manipulation of histone mobility is a unique chromatin regulatory mechanism adopted by alphaherpesviruses (EHV1 and HSV1) to directly regulate viral chromatin stability. Although individual aspects of EHV1 histone mobilization in equine cells are unique, histones are nonetheless most mobile within viral chromatin domains.

## Data Availability

All relevant data are available in the article, supplemental material, or from the corresponding author upon request.

## SUPPLEMENTAL MATERIAL

Supplemental material jvi.01589-25-s0001.pdf Figures S1 to S6 and Tables S1 to S3.

ASM does not own the copyrights to Supplemental Material that may be linked to, or accessed through, an article. The authors have granted ASM a non-exclusive, world-wide license to publish the Supplemental Material files. Please contact the corresponding author directly for reuse.
