# Supplementary material for: Equine histones are mobilized within equid alphaherpesvirus 1 (EHV1) replication compartments
Source: J Virol. 2025 Nov 25;99(12):e01589-25. doi: 10.1128/jvi.01589-25 (PMC12724232; doi:10.1128/jvi.01589-25)
Supplement: Supplemental material — Figures S1 to S6 and Tables S1 to S3. [file jvi.01589-25-s0001.pdf]

Figure Captions

Fig S1. Equine and human H2A.X, macroH2A, and H1.2 sequence comparison. Equine or human histone H2A.X (XP_023500737 or NP_002096, respectively), macroH2A (XP_023473434.1 or NP_001035248.1, respectively), and H1.2 (XP_005603676.1 or NP_005310, respectively) amino acid sequences pairwise aligned using MUSCLE Alignment in Geneious Prime 2025.1.2. Identical residues indicated by a dot. The conserved histone fold of core histones highlighted in pink; H2A docking domain highlighted in blue; macroH2A macro domain highlighted in green; conserved DNA-binding winged-helix motif of linker histones highlighted in brown. H2A.X residues phosphorylated in response to DNA damage (T136, S139) are conserved among equine and human and marked by red asterisks (1). Amino acid residues for highlighted regions as denoted for human histones on HistoneDB 2.0 (2).

Fig S2. Equine GFP-histones have much lower expression levels than endogenous histones. Whole cell lysates of EDerm cells transiently expressing GFP-H2B, -H3.3, or -H4. (**A**) Membrane probed with anti-GFP antibodies to detect GFP-histones. (**B**) The same membrane from (A) cut and probed with anti-H2B, -H3, or -H4 antibodies to detect endogenous and GFP-histones. Black arrows mark approximate molecular weight of GFP-H2B (41kDa), -H3.3 (42kDa), and -H4 (38kDa). Endogenous H2B, H3.3, and H4 are approximately 16kDa, 17kDa, and 11kDa, respectively. Black asterisk marks a cross-reacting antigen.

Fig S3. H3.1 is most dynamic in mock-infected cell chromatin, while H3.3 is most dynamic in "large" RCs. EDerm cells transfected with plasmids encoding GFP-H3.1 or -H3.3 were mock-infected or infected with 10 PFU/cell of non-neurotropic or neurotropic EHV1 at least 40h after transfection. GFP-H3.1 or -H3.3 nuclear mobility was evaluated by FRAP between 5 and 6hpi. FRAP data for each EHV1 infected cell were pooled for each histone and segregated by the presence (Large) or absence (Small) of clearly identifiable RCs, or diffuse H3 (Diffuse) (for examples see Fig 7B). (**A**) Line graphs present GFP-H3.1

(black) or -H3.3 (blue) FRAP. (**B**) Bar graph presents the average GFP-H3.3 normalized free pool expressed relative to the average GFP-H3.1 normalized free pool for the same compartment (set at 1). (**C**) Bar graph presents the average GFP-H3.3 initial normalized fluorescence recovery relative to the average GFP-H3.1 initial normalized fluorescence recovery for the same compartment (set at 1). Error bars, SEM; dashed error bars represent SEM for H3.1. H3.1 n≥46 cells per treatment from 5 independent experiments; H3.3 n≥60 cells per treatment from 6 independent experiments. ** *P*<0.01; * *P*<0.05; ns, not significant, Student's two-tailed T-test pairwise comparison of variant H3.3 to canonical H3.1 for each.

Fig S4. Variant H2A histones are differentially mobilized in RCs. EDerm cells transfected with plasmids encoding GFP-H2A, -H2A.Z, -H2A.X, -macroH2A, or -H2A.B were mock-infected or infected with 10 PFU/cell of non-neurotropic or neurotropic EHV1 at least 40h after transfection. GFP-H2A, -H2A.Z, -H2A.X, -macroH2A, or -H2A.B nuclear mobility was evaluated by FRAP between 5 and 6hpi. Data for each EHV1 infected cell were pooled for each histone and segregated by the presence (Large) or absence (Small) of identifiable RCs for histones H2A, H2A.Z, H2A.X, or macroH2A (for examples see Fig 9B), or by mobility group for histone H2A.B (for examples see Fig 11B). (**A**) Line graphs present GFP-H2A, -H2A.Z, -H2A.X, or -macroH2A FRAP. (**B**) Bar graphs present the average normalized free pool of GFP-H2A.Z, -H2A.X, -macroH2A, or -H2A.B mobility groups 1 to 4+ expressed relative to the average normalized GFP-H2A free pool for the same compartment (set at 1). (**C**) Bar graphs present the average initial normalized fluorescence recovery for GFP-H2A.Z, -H2A.X, -macroH2A, or -H2A.B mobility groups 1 to 4+ expressed relative to the average GFP-H2A initial normalized fluorescence recovery for the same compartment (set at 1). (**B, C**) H2A.B mobility groups 1 to 4+ in EHV1-infected cells expressed relative to the H2A average in infected-cell chromatin or "large" RC for comparison. Error bars, SEM. n≥38 cells per treatment from 4 independent experiments. ** *P*<0.01; * *P*<0.05; ns, not significant, Student's two-tailed T-test for pairwise comparison of variant H2A to canonical H2A for each.

Fig S5. Canonical and variant H2A or H1.2 free pools do not relate to GFP-histone expression levels. EDerm cells transfected with plasmids encoding GFP-H2A, -H2A.Z, -H2A.X, -macroH2A, -H2A.B, or -H1.2 were mock-infected or infected with 10 PFU/cell of non-neurotropic or neurotropic EHV1 at least 40h after transfection. GFP-histone nuclear mobility was evaluated by FRAP between 5 and 6hpi. FRAP data for each EHV1 infected cell was pooled for each histone and segregated by the presence (Large) or absence (Small) of clearly identifiable RCs for histones H2A, H2A.Z, H2A.X, macroH2A, and H1.2 or by mobility group for H2A.B. Dot plots present the level of free (**A**) GFP-H2A, -H2A.Z, -H2A.X, -macroH2A, (**B**) -H2A.B, or (**C**) H1.2 per individual cell plotted against its normalized total nuclear fluorescence before photobleaching. n≥38 cells per treatment from 4 independent experiments.

Fig S6. H2A.B is variably mobilized in non-nucleolar chromatin. EDerm cells transfected with plasmids encoding GFP-H2A.B were mock-infected or infected with 10 PFU/cell of non-neurotropic or neurotropic EHV1 at least 40h after infection. GFP-H2A.B nuclear mobility was evaluated by FRAP between 5 and 6hpi. FRAP data for mock-infected cell chromatin or EHV1-infected cells were pooled and segregated by mobility group. (**A**) Line graphs present average GFP-H2A.B FRAP per mobility group in mock- or EHV1-infected cells. (**B**) GFP-H2A.B FRAP within each individual photobleached region of EHV1-infected or mock-infected cell chromatin. (**C**) GFP-H2A.B FRAP within each individual photobleached region of mock-infected nucleoli. Error bars, SEM. n≥38 per treatment cells from 4 independent experiments.

H2A.X

Histone Fold — Docking Domain

```
                  10         20         30         40         50         60         70         80         90        100
Consensus  MSGRGKTGGK ARAKAKSRSS RAGLQFPVGR VHRLLRKGHY AERVGAGAPV YLAAVLEYLT AEILELAGNA ARDNKKTRII PRHLQLAIRN DEELNKLLGG
   Equine  .......... .......... .......... .......... .......... .......... .......... .......... .......... ..........
    Human  .......... .......... .......... .......... .......... .......... .......... .......... .......... ..........

                 110        120        130        140
Consensus  VTIAQGGVLP NIQAVLLPKK TSATVGPKAP XGGKKATQAS QEY
   Equine  .......... .......... .......... A......... ...
    Human  .......... .......... .......... S......... ...
                                                *   *
```

macroH2A

Histone Fold — Docking Domain

```
                  10         20         30         40         50         60         70         80         90        100
Consensus  MSSRGGKKKS TKTSRSAKAG VIFPVGRMLR YIKKGHPKYR IGVGAPVYMA AVLEYLTAEI LELAGNAARD NKKGRVTPRH ILLAVANDEE LNQLLKGVTI
   Equine  .......... .......... .......... .......... .......... .......... .......... .......... .......... ..........
    Human  .......... .......... .......... .......... .......... .......... .......... .......... .......... ..........

                 110        120        130        140        150        160        170        180        190        200
Consensus  ASGGVLPNIH PELLAKKRGS KGKLEAIIXP PPAKKAKSPS QKKPVSKKAG GKKGARKSKK QGEVSKAASA DSTTEGTPAD GFTVLSTKSL FLGQKLNLIH
   Equine  .......... .......... ........M. .......... .......... .......... .......... .......... .......... ..........
    Human  .......... .......... ........T. .......... .......... .......... .......... .......... .......... ..........
```

Macro Domain

```
                 210        220        230        240        250        260        270        280        290        300
Consensus  SEISNLAGFE VEAIINPTNA DIDLKDDLGN TLEKKGGKEF VEAVLELRKK NGPLEVAGAA XSAGHGLPAK FVIHCNSPVW GXDKCEELLE KTVKNCLALA
   Equine  .......... .......... .......... .......... .......... .......... L......... .......... .V........ ..........
    Human  .......... .......... .......... .......... .......... .......... V......... .......... .A........ ..........

                 310        320        330        340        350        360        370
Consensus  DDKKLKSIAF PSIGSGRNGF PKQTAAQLIL KAISSYFVST MSSSIKTVYF VLFDSESIGI YVQEMAKLDA N
   Equine  .......... .......... .......... .......... .......... .......... .......... .
    Human  .......... .......... .......... .......... .......... .......... .......... .
```

H1.2

Winged Helix Motif

```
                  10         20         30         40         50         60         70         80         90        100
Consensus  MSETAPAAPA AAPPAEKAPV KKKAXKKKXG XXRKASGPPV SELITKAVAA SKERSGVSLA ALKKALAAAG YDVEKNNSRI KLGLKSLVSK GTLVQTKGTG
   Equine  .......... .......... ....T..PA. AR........ .......... .......... .......... .......... .......... ..........
    Human  .......... .......... ....A..AG. TP........ .......... .......... .......... .......... .......... ..........

                 110        120        130        140        150        160        170        180        190        200
Consensus  ASGSFKLNKK AASGEAKPKX KKXGXXKPKK PXGAAKKPKK AXGXATPKKS AKKTPKKAKK PAAAXVTKKV AKSPKKAKXA KPKKAAKSAX KAVKPKAAKP
   Equine  .......... .........A ..T.AA.... .A........ .T.A...... .......... ....A..... ........T. .........P ..........
    Human  .......... .........V ..A.GT.... .V........ .A.G...... .......... ....T..... ........V. .........A ..........

                 210
Consensus  KVXKPKKAAP KKK
   Equine  ..A....... ...
    Human  ..V....... ...
```

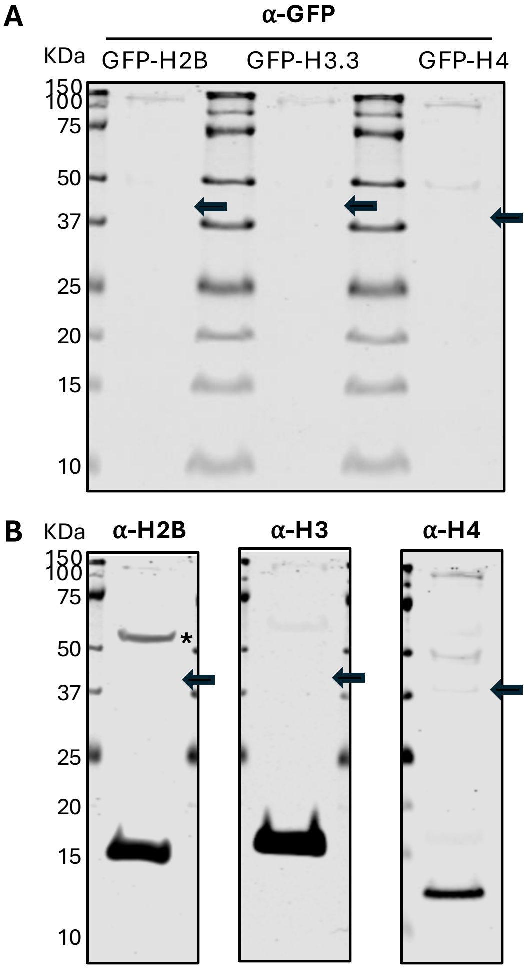
A
α-GFP
KDa
GFP-H2B
GFP-H3.3
GFP-H4
150
100
75
50
37
25
20
15
10
B
KDa
α-H2B
α-H3
α-H4
150
100
75
50
37
25
20
15
10
*

Fig S3

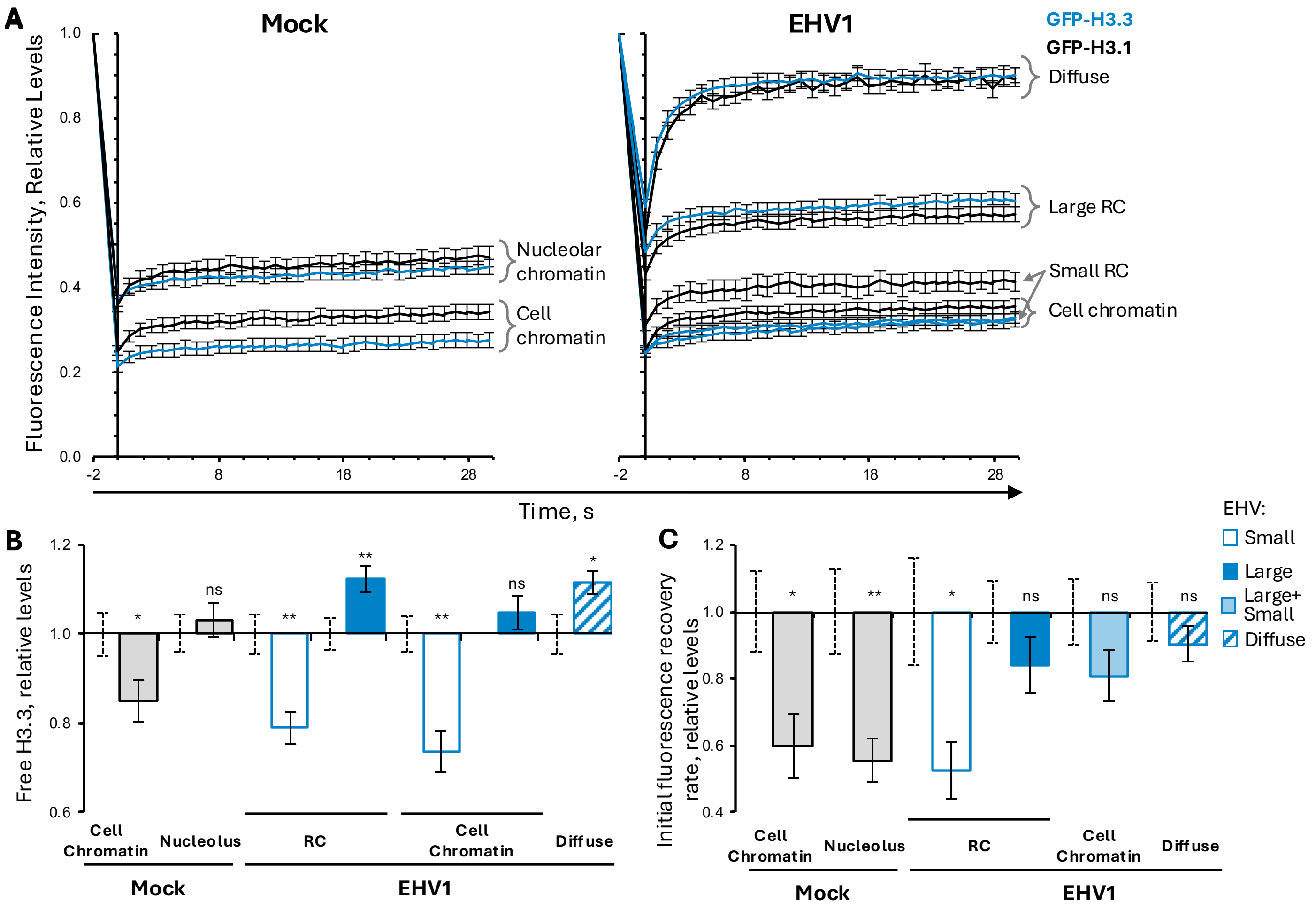
A
Mock
EHV1
GFP-H3.3
GFP-H3.1
Diffuse
Large RC
Small RC
Cell chromatin
Nucleolar chromatin
Cell chromatin
Fluorescence Intensity, Relative Levels
1.0
0.8
0.6
0.4
0.2
0.0
-2
8
18
28
Time, s
B
Free H3.3, relative levels
1.2
1.0
0.8
0.6
*
ns
**
**
**
ns
*
Cell Chromatin
Nucleolus
RC
Cell Chromatin
Diffuse
Mock
EHV1
C
Initial fluorescence recovery rate, relative levels
1.2
1.0
0.8
0.6
0.4
*
**
*
ns
ns
ns
Cell Chromatin
Nucleolus
RC
Cell Chromatin
Diffuse
Mock
EHV1
EHV:
Small
Large
Large+ Small
Diffuse

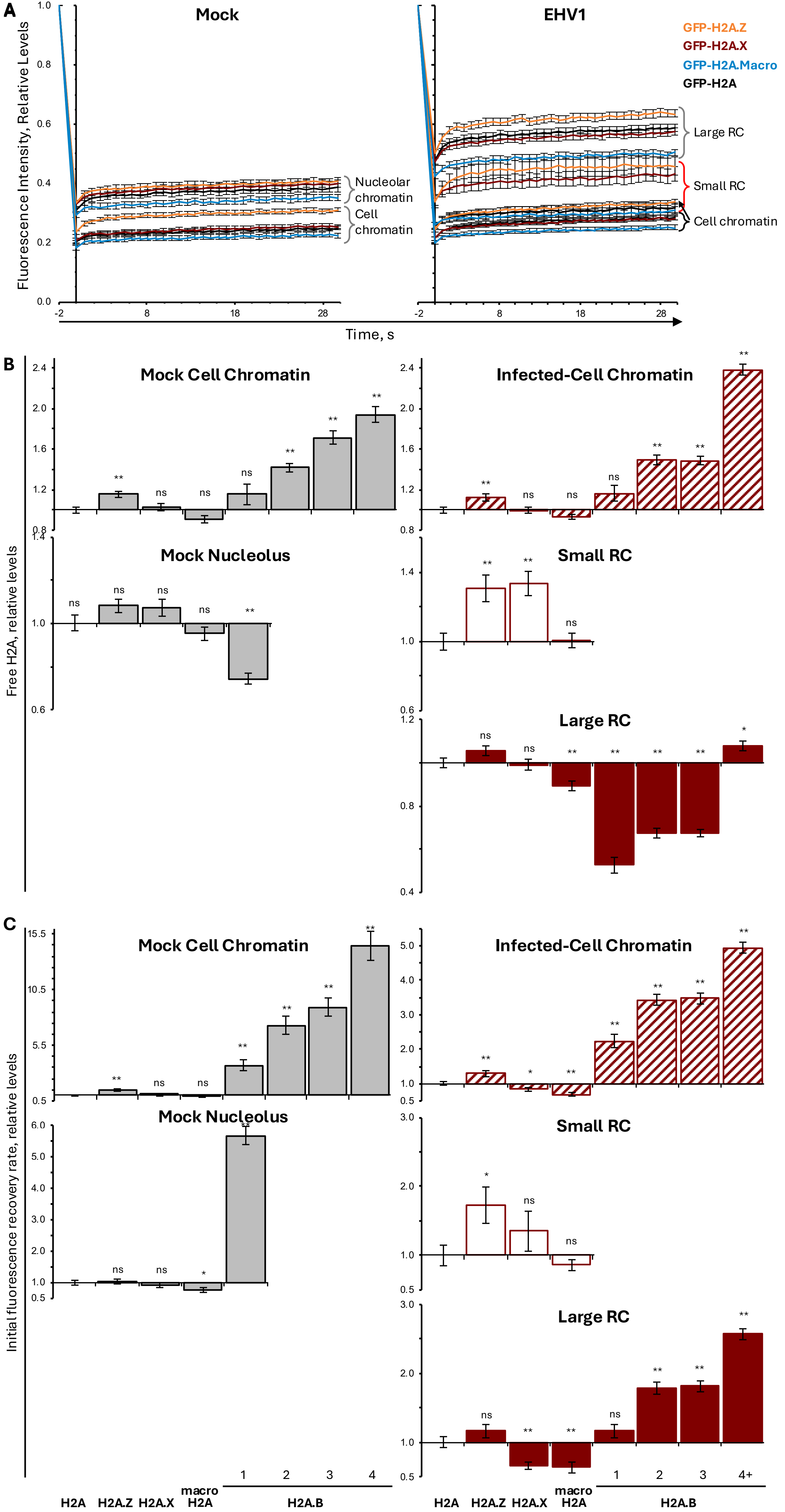
A
Mock
EHV1
GFP-H2A.Z
GFP-H2A.X
GFP-H2A.Macro
GFP-H2A
Fluorescence Intensity, Relative Levels
Nucleolar chromatin
Cell chromatin
Large RC
Small RC
Cell chromatin
Time, s
B
Mock Cell Chromatin
Infected-Cell Chromatin
Mock Nucleolus
Small RC
Large RC
Free H2A, relative levels
C
Mock Cell Chromatin
Infected-Cell Chromatin
Mock Nucleolus
Small RC
Large RC
Initial fluorescence recovery rate, relative levels
H2A
H2A.Z
H2A.X
macro H2A
H2A.B
1
2
3
4
4+

Fig S5

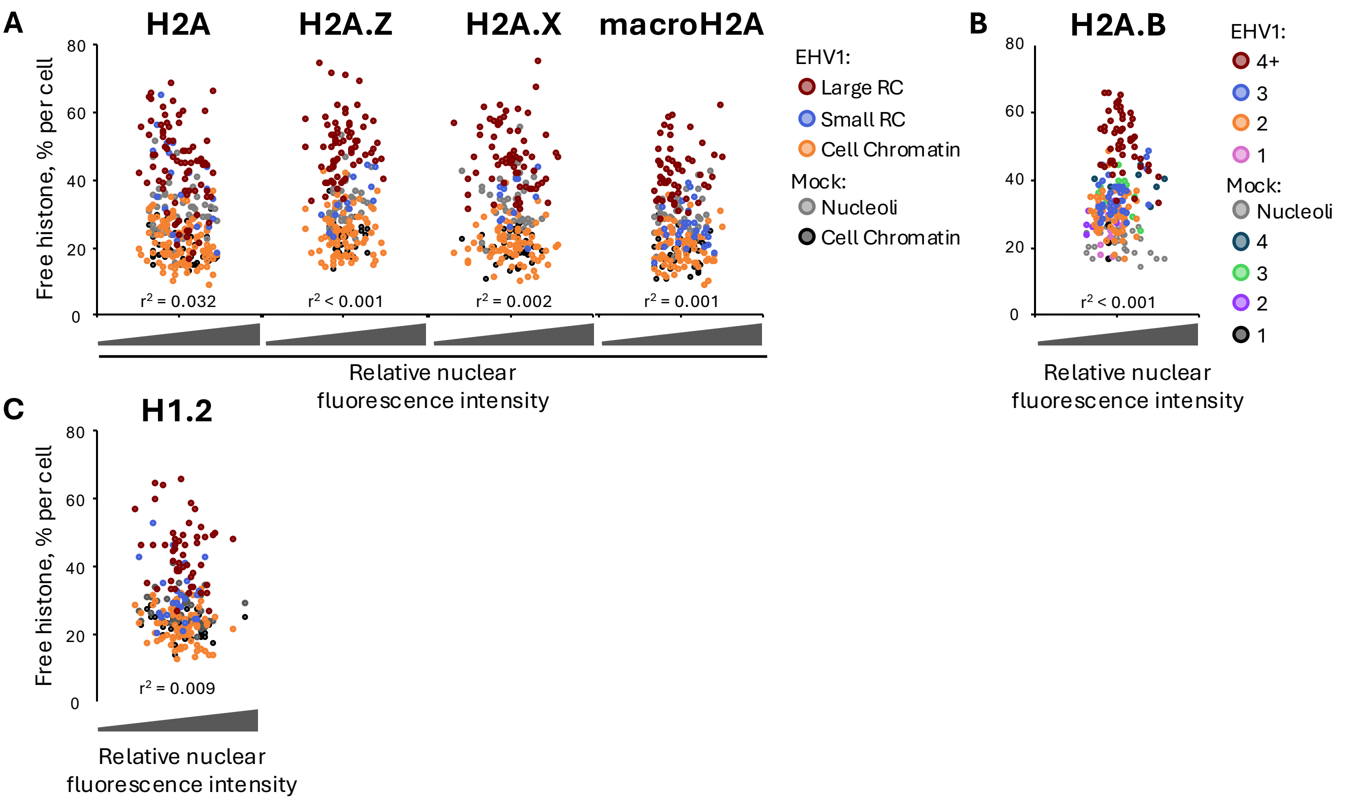

A
H2A
H2A.Z
H2A.X
macroH2A
Free histone, % per cell
80
60
40
20
0
r² = 0.032
r² < 0.001
r² = 0.002
r² = 0.001
Relative nuclear fluorescence intensity
EHV1:
Large RC
Small RC
Cell Chromatin
Mock:
Nucleoli
Cell Chromatin
B
H2A.B
r² < 0.001
Relative nuclear fluorescence intensity
EHV1:
4+
3
2
1
Mock:
Nucleoli
4
3
2
1
C
H1.2
r² = 0.009
Relative nuclear fluorescence intensity

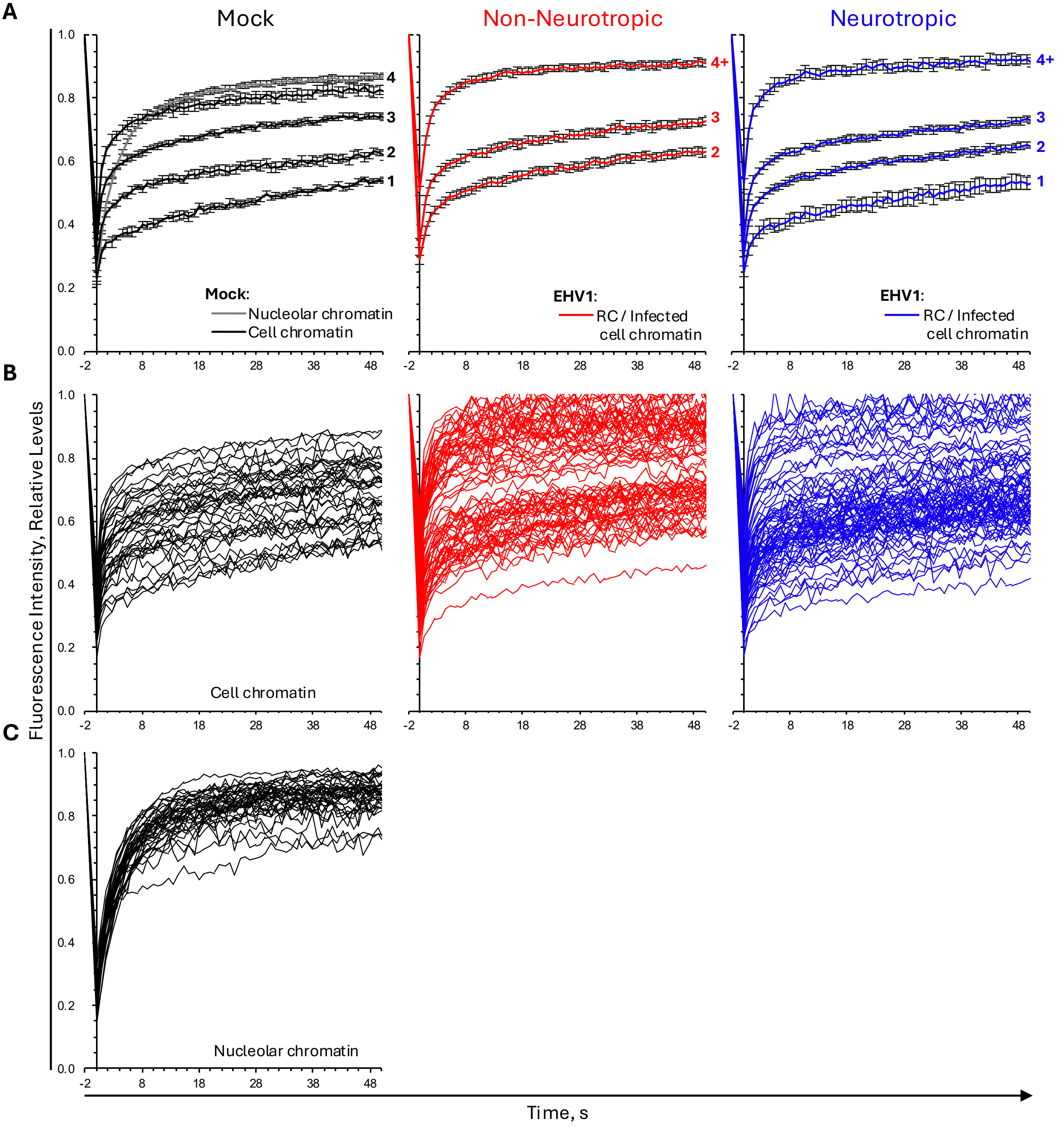

A
Mock
Non-Neurotropic
Neurotropic
Mock:
Nucleolar chromatin
Cell chromatin
EHV1:
RC / Infected cell chromatin
4
3
2
1
4+
B
Cell chromatin
C
Nucleolar chromatin
Fluorescence Intensity, Relative Levels
Time, s
1.0
0.8
0.6
0.4
0.2
0.0
-2
8
18
28
38
48

Table S1. Percent of EHV1-infected cells with "small" or "large" RCs.

		Small	Large	Diffuse
H2B	NN[a]	24	76	
	N[b]	30	70	
H4	NN[a]	23	77	
	N[b]	33	67	
H3.1	NN[a]	11	71	18
	N[b]	21	67	12
H3.3	NN[a]	26	54	20
	N[b]	16	59	25
H2A	NN[a]	29	71	
	N[b]	13	87	
H2A.Z	NN[a]	8	92	
	N[b]	23	77	
H2A.X	NN[a]	15	85	
	N[b]	20	80	
Macro H2A	NN[a]	30	70	
	N[b]	38	62	
H1.2	NN[a]	26	74	
	N[b]	39	61	

[a] Non-Neurotropic

[b] Neurotropic

Table S2. Variant histone mobilities expressed relative to canonical histone mobility.

					Free Histone			Fast Recovery Rate			Slow Recovery Rate		
					Relative Level,% (avg ± SEM)	*P*	Cells with large increase[a], %	Relative Level,% (avg ± SEM)	*P*	Cells with large increase[a], %	Relative Level,% (avg ± SEM)	*P*	Cells with large increase[a], %
H3.3	Mock	Chromatin	H3.1		100 ± 5	-	20	100 ± 12	-	13	100 ± 20	-	13
			H3.3		85 ± 5	*	39[b]	60 ± 10	*	27[b]	75 ± 8	ns	0[b]
		Nucleolus	H3.1		100 ± 4	-	17	100 ± 13	-	15	100 ± 21	-	11
			H3.3		103 ± 4	ns	17	56 ± 7	**	18[b]	120 ± 13	ns	6
	EHV	Cell Chromatin	H3.1		100 ± 4	-	18	100 ± 10	-	13	100 ± 11	-	13
			H3.3 small		73 ± 5	**	35[b]	81 ± 8	ns	2[b]	128 ± 14	ns	19
			H3.3 large		105 ± 4	ns	14						
		Small RC	H3.1		100 ± 4	-	13	100 ± 16	-	14	100 ± 30	-	10
			H3.3		79 ± 4	**	58[b]	52 ± 8	*	46[b]	275 ± 64	*	48
		Large RC	H3.1		100 ± 3	-	19	100 ± 9	-	20	100 ± 15	-	16
			H3.3		113 ± 3	**	30	84 ± 9	ns	17[b]	135 ± 22	ns	20
		Diffuse	H3.1		100 ± 4	-	21	100 ± 9	-	11	100 ± 14	-	14
			H3.3		112 ± 3	*	28	90 ± 5	ns	17	64 ± 9	*	45[b]
H2A	Mock	Chromatin	H2A		100 ± 3	-	19	100 ± 8	-	11	100 ± 9	-	16
			H2A.Z		116 ± 3	**	38	144 ± 12	**	35	116 ± 10	ns	25
			H2A.X		102 ± 3	ns	23	102 ± 7	ns	13	99 ± 14	ns	18
			MacroH2A		91 ± 3	ns	28[b]	95 ± 10	ns	21[b]	110 ± 16	ns	15
			H2A.B	1	115 ± 11	ns	40	371 ± 54	**	100			
				2	142 ± 4	**	83	728 ± 82	**	100			
				3	171 ± 7	**	100	894 ± 83	**	100			
				4	194 ± 7	**	100	1442 ± 131	**	100			
		Nucleolus	H2A		100 ± 3	-	11	100 ± 8	-	15	100 ± 11	-	12
			H2A.Z		107 ± 3	ns	15	101 ± 7	ns	8	121 ± 13	ns	26
			H2A.X		107 ± 4	ns	23	92 ± 8	ns	15[b]	116 ± 13	ns	30
			MacroH2A		95 ± 3	ns	15[b]	76 ± 7	*	16[b]	139 ± 18	ns	36
			H2A.B		74 ± 2	**	56[b]	567 ± 30	**	100			
	EHV	Cell Chromatin	H2A		100 ± 3	-	19	100 ± 5	-	19	100 ± 8	-	14
			H2A.Z		113 ± 3	**	28	131 ± 9	**	27	130 ± 9	*	20
			H2A.X		99 ± 3	ns	13[b]	84 ± 5	*	18[b]	103 ± 12	ns	8
			MacroH2A		93 ± 3	ns	16[b]	69 ± 5	**	39[b]	86 ± 7	ns	5[b]
			H2A.B	1	116 ± 8	ns	30	224 ± 19	**	90			
				2	149 ± 5	**	78	342 ± 16	**	98			
				3	149 ± 4	**	80	348 ± 16	**	95			
				4+	238 ± 5	**	100	493 ± 16	**	100			
		Small RC	H2A		100 ± 5	-	20	100 ± 15	-	16	100 ± 14	-	22
			H2A.Z		131 ± 8	**	58	172 ± 26	*	45	69 ± 18	ns	44[b]
			H2A.X		133 ± 7	**	64	136 ± 29	ns	21	144 ± 35	ns	43
			MacroH2A		101 ± 4	ns	19	86 ± 8	ns	8[b]	115 ± 20	ns	21
		Large RC	H2A		100 ± 2	-	17	100 ± 8	-	12	100 ± 10	-	16
			H2A.Z		105 ± 2	ns	18	117 ± 9	ns	20	163 ± 17	**	28
			H2A.X		99 ± 2	ns	18	66 ± 6	**	22[b]	99 ± 11	ns	17
			MacroH2A		89 ± 2	**	30[b]	64 ± 8	**	32[b]	117 ± 14	ns	17
			H2A.B	1	53 ± 4	**	100[b]	117 ± 10	ns	10			
				2	67 ± 2	**	85[b]	179 ± 8	**	59			
				3	67 ± 2	**	91[b]	182 ± 8	**	57			
				4+	108 ± 2	*	27	257 ± 8	**	96			

[a] percentage of cells > 1SD above average value for the canonical histone.

[b] percentage of cells > 1SD below average value for the canonical histone.

P values represent Student's two-tailed T-test pairwise comparison of canonical and variant histone mobilities.

***P*<0.01, **P*<0.05, ns = not significantly different.

Table S3. Percent of cells per H2A.B mobility group.

Mobility Group	Mock	EHV1	
		Non-Neurotropic	Neurotropic
1	13	0	13
2	31	32	37
3	38	21	29
4/4+	18	47	21

References

1. Li A, Yu Y, Lee S-C, Ishibashi T, Lees-Miller SP, Ausió J. 2010. Phosphorylation of histone H2A.X by DNA-dependent protein kinase is not affected by core histone acetylation, but it alters nucleosome stability and histone H1 binding. J Biol Chem 285:17778–88.

2. Draizen EJ, Shaytan AK, Mariño-Ramírez L, Talbert PB, Landsman D, Panchenko AR. 2016. HistoneDB 2.0: a histone database with variants--an integrated resource to explore histones and their variants. Database (Oxford) 2016:baw014.
